# Simulation study comparing analytical methods for single-item longitudinal patient-reported outcomes data

**DOI:** 10.1007/s11136-022-03267-z

**Published:** 2022-10-17

**Authors:** Vinicius F. Calsavara, Márcio A. Diniz, Mourad Tighiouart, Patricia A. Ganz, N. Lynn Henry, Ron D. Hays, Greg Yothers, André Rogatko

**Affiliations:** 1 Cedars-Sinai Medical Center, Samuel Oschin Comprehensive Cancer Institute, Los Angeles, CA USA; 2 University of California Los Angeles Jonsson Comprehensive Cancer Center, Los Angeles, CA USA; 3 University of Michigan Rogel Cancer Center, Ann Arbor, MI USA; 4 Department of Medicine, University of California Los Angeles, Los Angeles, CA USA; 5 University of Pittsburgh, Pittsburgh, PA USA

**Keywords:** Adverse event, Cumulative logit mixed model, Probabilistic index model, Patient-reported outcome, Type I and II errors, PRO-CTCAE

## Abstract

**Purpose:**

Efficient analytical methods are necessary to make reproducible inferences on single-item longitudinal ordinal patient-reported outcome (PRO) data. A thorough simulation study was performed to compare the performance of the semiparametric probabilistic index models (PIM) with a longitudinal analysis using parametric cumulative logit mixed models (CLMM).

**Methods:**

In the setting of a control and intervention arm, we compared the power of the PIM and CLMM to detect differences in PRO adverse event (AE) between these groups using several existing and novel summary scores of PROs. For each scenario, PRO data were simulated using copula multinomial models. Comparisons were also exemplified using clinical trial data.

**Results:**

On average, CLMM provided substantially greater power than the PIM to detect differences in PRO-AEs between the groups when the baseline-adjusted method was used, and a small advantage in power when using the baseline symptom as a covariate.

**Conclusion:**

Although the CLMM showed the best performance among analytical methods, it relies on assumptions difficult to verify and that might not be fulfilled in the real world, therefore our recommendation is the use of PIM models with baseline symptom as a covariate.

**Supplementary Information:**

The online version contains supplementary material available at 10.1007/s11136-022-03267-z.

## Introduction

In 2016, Congress passed the 21st Century Cures Act, authorizing $1.8 billion in funding for the Cancer Moonshot over 7 years. This effort brought together a large community of investigators and clinicians dedicated to expedite research to improve the lives of people with cancer and their loved ones. A Blue Ribbon Panel was assembled to make recommendations related to high priority areas for research funding, and one of the areas identified was the acceleration of research that can identify approaches to monitor and manage patient-reported symptoms [1]. This goal has been addressed in part through an NIH funding announcement in 2017 whose stated purpose was to stimulate the development of methods for better understanding the treatment tolerability by analyzing data from clinical trials using adverse event data through the use of the Common Terminology Criteria for Adverse Events (CTCAE), the Patient-Reported Outcome (PRO) version of the CTCAE (PRO-CTCAE™), and other clinically relevant data (e.g., stage of disease, laboratory findings, co-morbidities, concurrent medications).

CTCAE data that are assessed by the clinical investigator reflect diverse sources of data: laboratory studies, physical examination findings, as well as symptoms elicited during history-taking by the clinician. CTCAE data are usually collected after exposure to treatment (e.g., after the first cycle of treatment and subsequent cycles, or at timed intervals such as every three months). In contrast, PRO data reflect the patient’s own assessment of a symptom or toxicity (e.g., pain, fatigue, nausea), and are generally collected prior to treatment (baseline), and then at some regular interval after treatment initiation. The development of the PRO-CTCAE item bank [2] has been a major addition to the toolbox for assessment cancer treatment toxicity, along with existing symptom measures used to assess cancer treatment-related toxicities (e.g., MD Anderson Symptom Inventory [3], the Edmonton Symptom Assessment System [4], Patient-Reported Outcomes Measurement Information System [5]). PRO data complement CTCAE data, especially when a pre-treatment assessment is included. In addition, clinicians’ ratings of symptoms or toxicities often underestimate the severity of the symptom [2, 6].

Traditionally, longitudinal CTCAE data have been summarized and reported as the maximum (Max) toxicity grade experienced by patients receiving a particular treatment. In our work to date, we have shown that an alternative summary, the Toxicity Index (TI) [7], has greater power to detect differences between treatments than max and average (Avg) as summary measures [8]. Moreover, we have shown that summary measures modeled using a semiparametric regression model, the probabilistic index model (PIM), allow incorporation of patients’ characteristics in the model, increasing the understanding of toxicity as recently reported in two separate clinical trials [8, 9]. The TI may be particularly valuable when considering cancer therapies that are taken chronically, and where patients may be at risk for discontinuation due to the accumulation of multiple low-grade toxicities.

Longitudinal data analysis of PROs is traditionally performed considering an overall measure (e.g., sum, average) for a group of single items over time using standard regression models such as linear mixed models (LMM) [10, 11]. However, there is no standard approach for the analysis of PRO-CTCAE items, which vary from 0 to 4 that can be interpreted by themselves and they are not summarized into an overall measure. Publications to date related to the analysis of PRO-CTCAE items have been analyzed as the proportion of scores greater than a given cut-off value (e.g., score ≥ 3) with comparisons between treatments based on the Fisher’s exact test or chi-squared test [12–14]. To incorporate the baseline PRO-CTCAE score in those analyses, a modified version of this approach has been proposed based on the change from baseline recording the maximum scores only for those scores that were worse than the baseline grade [12, 15, 16]. Recently, this approach to adjust by baseline was extended when calculating TI with comparisons between treatments based on the Wilcoxon rank-sum test [15]. Nonetheless, these methods do not allow incorporation of patients’ covariates in the analysis of PRO-CTCAE items and modeling the change has been shown as statistically inefficient [17].

In this manuscript, we analyze PRO single items—such as PRO-CTCAE items—longitudinally using the cumulative logit mixed model (CLMM) as a direct extension of the LMM used for traditional PRO outcomes. We also summarize repeated single items over time with commonly used summary measures in CTCAE data—TI, Max and Avg—with the baseline score items incorporated following two modeling strategies: (i) calculating the change from baseline and (ii) considering baseline as a covariate. Simulation studies were used to compare these methods in terms of type I and II error rates in testing the presence of treatments effect. No study to date has compared the performance of these approaches for single items that are ordinal longitudinal data. To reflect the reality of most clinical trials, the generated datasets were also simulated according to the observed data from the randomized, double-blind NSABP B-35 clinical trial [18].

## Methods

### Clinical trial data

The case study consists of individual patient data from NSABP B-35, a Phase III double-blind randomized, placebo-controlled trial comparing 5 years of treatment with tamoxifen versus anastrozole in postmenopausal women with hormone receptor-positive ductal carcinoma in situ [19]. Patient-reported symptom data were collected at baseline and every 6 months after treatment initiation, and a list of predefined 30 CTCAEs was selected to evaluate the patient’s toxicity experience during the trial. Each item was scored from 0 (least severe) to 4 (most severe). The CTCAE data provide detailed longitudinal information about the severity and types of toxicity. The trial included 3104 patients, with patient-reported symptom data available for the first 1194 patients. The statistical analysis methods were applied to three time points (baseline, and 6- and 12 months during therapy) for each CTCAE. Additional details of the trial are reported elsewhere [18, 19]. Observed proportions for each AE category by arm over time are summarized in the Online Supplement Section 1.

We briefly review the statistical methods used in the analysis of the clinical trial data and the simulation study for comparing toxicity between arms. Table 1 illustrates statistical analysis methods for single-item longitudinal data.

**Table 1:** Analysis methods for single-item longitudinal data

Score aggregated	Summary measure^c^	Method to summarize in a single measure	Selected scores^a^	Fitted model	Baseline as covariate in fitted model	Comparison between arms performed with statistical test	Statistical analysis method
Yes	TI	Baseline-adjusted	Follow-up scores that had at least a 1-score increase in AE score from baseline^b^	PIM	No	Wald test	PIM Baseline-adjusted TI
	Avg						PIM Baseline-adjusted Avg
	Max						PIM Baseline-adjusted Max
	TI	Post-baseline	Only follow-up scores	PIM	Yes	Wald test	PIM Baseline as covariate TI
	Avg						PIM Baseline as covariate Avg
	Max						PIM Baseline as covariate Max
No	None	No method applied	Only follow-up scores	CLMM	Yes	LRT	CLMM
						LRT by parametric bootstrap	CLMM (Bootstrap)

^a^Denotes how each method summarizes scores into a single value

^b^If the follow-up scores are less than or equal to the baseline score, then the summary measure is zero

^c^Summary measure is computed based on selected scores. LRT denotes likelihood ratio test

### Repeated measures and incorporating baseline items

Each longitudinal single item can be analyzed considering two general approaches: (i) describe single items as ordinal repeated scores using an appropriate mixed regression model; (ii) summarize single items into a single measure, then apply an appropriate regression model for the single measure as the response variable. Several single measures have been proposed for the CTCAE data analysis—TI, Avg, and Max—therefore, we consider them as candidates as summary indexes for PRO data analysis as well.

In both approaches, baseline item can be incorporated following two reasonings: (a) as a covariate with post-baseline toxicity scores as the response variable; and (b) calculating a change in toxicity from baseline, denoted baseline-adjusted following previous work [12, 15, 16], such that change in toxicity score is the response variable. In this framework, toxicity summary indexes can be calculated either considering post-baseline scores and baseline-adjusted scores. Mathematical definitions of TI, Max, and Avg for post-baseline and baseline-adjusted scores with calculations are presented in the Online Supplement Section 2.

### Probabilistic index model (PIM)

When summarizing repeated measures into a single score such as TI and Max, standard regression models may not be appropriate, and therefore, rank-based methods are applied. The PIMs are a class of semiparametric regression models [8, 20–24] in which the probability index (PI) is modeled as a function of covariates. The PI is a relative effect measure that corresponds to the probability that the outcome (e.g., toxicity) of a patient randomly sampled from group 1 is greater than the outcome of another patient randomly sampled from group 0, conditional on the covariate values of both patients [25]. In other words, let ${S}_{0}$ and ${S}_{1}$ be the random variables representing toxicity scores for groups 0 and 1, respectively: when PI is statistically significantly greater than 0.5 (i.e., PI=$P\left({S}_{0}<{S}_{1}\right)>$0.5), then patients in group 0 are more likely to have lower scores compared to patients in group 1; when PI is statistically significantly less than 0.5, then patients in group 0 are more likely to have higher scores than in group 1, and a PI equal to 0.5 means that both groups have similar toxicity score distribution.

In the single-item longitudinal data analysis, the PIM was fitted considering the toxicity burden summaries—TI, Avg, and Max—for (a) post-baseline scores as the response variable with baseline score and treatment as covariates (denoted as PIM Baseline as covariate) and (b) baseline-adjusted scores as the response variable with treatment as a covariate (denoted as PIM Baseline-adjusted). The PI with Wald-type 95% confidence interval was reported with p values based on the Wald statistic.

### Cumulative logit mixed model (CLMM)

The CLMM allows modeling ordinal-scale data, i.e., observations that fall in an ordered finite set of categories, while taking into account the correlation structure of the data over time. It is a powerful model for such data since observations are treated correctly as categorical but the ordered nature of the data is exploited [26–28].

Although the CLMM model is appropriate to analyze ordinal-scale data over time, tests for statistical significance are problematic for both fixed and random effects. The likelihood ratio tests (LRT) are widely used to test fixed effects, but these tests can result in significant p values when the null hypothesis is true [29]. The LRT is usually appropriate for inference about random effects, but corrections are needed to address boundary problems [30, 31]. The reliable inferences for fixed and random effects in generalized linear mixed models can be done using alternative methods such as parametric bootstrap. Rather than relying on a chi-square distribution, the distribution of the LR statistic is constructed using a parametric bootstrap approach, which does not make any assumptions about degrees of freedom and the p value is estimated by using repeated sampling [32]. It is computationally intensive and requires longer run time than the usual LRT, but allows for better control of the type I error.

Longitudinal data analysis was performed with CLMM using maximum likelihood estimation with Laplace approximation. Each CLMM included the follow-up scores as a dependent variable, the intercept, an independent variable representing time points, baseline measurement and treatment variable as covariates, and a random patient effect. Odds ratios along with 95% confidence intervals (95%CI) are reported. The *p* value associated with the treatment effect was obtained by using the usual LRT and LRT by parametric bootstrap with 500 bootstrap resamples (see the Online Supplement Section 3 for a description of the CLMM and algorithm's implementation to perform the LRT by parametric bootstrap).

Throughout this article, we will denote CLMM and CLMM (Bootstrap) as the statistical analysis methods when LRT and LRT by parametric bootstrap were applied to single-item longitudinal data analysis, respectively.

### Simulation studies

We conducted simulation studies based on a two-arm randomized-controlled design with ordinal response to mimic the B-35 trial adverse events data. Without loss of generality, we assume that both arms contain an equal number of 100 patients. The simulation studies were carried out considering only a single adverse event for each patient with five different ordinal categories (ranging from 0–4) evaluated at three time points. Correlated responses were generated using a copula multinomial model [33] by fixing the marginal probabilities for the categories at each time point and the correlation among the repeated measures in each arm.

For each generated dataset, the comparison between arms was performed using the following eight statistical analysis methods: (1 PIM Baseline-adjusted (1a) TI, (1b) Avg, and (1c) Max; 2) PIM Baseline as covariate (2a) TI, (2b) Avg, and (2c) Max; 3a) CLMM and 3b) CLMM (Bootstrap). For each scenario, 1000 independent datasets were generated to estimate the type I and II error rates (power = 1-type II error).

All simulations and analyses were conducted using the R software version 4.0 [34] with packages pim [35] and ordinal [36]. All hypotheses were two-tailed with 5% significance level. If the generated sample did not present data in at least two categories over time or any of these fitted models did not converge (i.e., to find a solution) within a reasonable number of iterations, the sample was to be redrawn. Thus, the simulation results are not influenced by numerical convergence issues. The convergence time of CLMM statistical analysis method tends to increase with the complexity of the model, especially the random effects structure. The CLMM (Bootstrap) analysis method is computationally intensive and requires longer run times, and so 500 resamples bootstrap was chosen to make the simulations computationally feasible. The results of the analysis methods for each simulated scenario took about 10 h of total CPU time using an Intel(R) Xeon(R) Gold 2.90 GHz [32 CPUs], 32 GB RAM, and Microsoft Windows 10 Enterprise.

## Results

We briefly illustrate the results of a single-item longitudinal PRO data analysis (headache) from the B-35 trial using the statistical analysis methods aforementioned.

Furthermore, we report the results of the estimated type I error rates and power estimates obtained in the simulation study. Type I error rate is estimated as the proportion of rejection of the null hypothesis (*H*_0_) when it is true, i.e., when there is not difference between arms, and the power is computed as the proportion of rejection of the when it is false, i.e., when the two groups are different over time.

### Clinical trial data

The violin plots display the patient-reported headache symptom distribution summarized by baseline-adjusted and post-baseline methods, where higher headache score was observed for anastrozole treatment when the post-baseline method is applied, while for the baseline-adjusted method, the summary measure distribution shape was close in both treatments (see the Online Supplement Section 4).

**Fig. 1:**
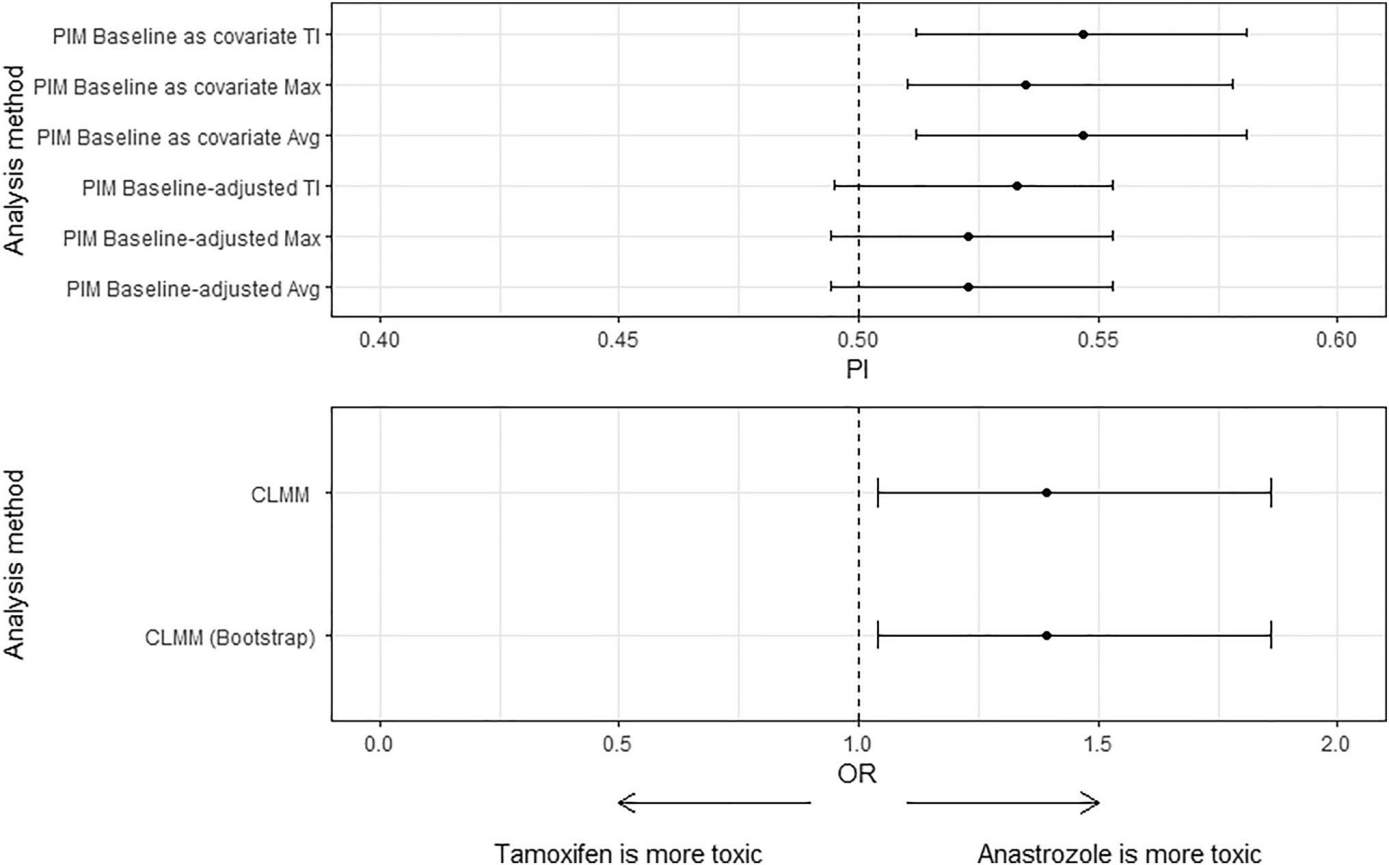
Forest plots demonstrating results of PIM and CLMM for the headache. If the effect size (probability index) is less than 0.5, then probability of the toxicity for Tamoxifen is greater than Anastrozole is small, indicating that Anastrozole has higher headaches score. Odds ratio of the event $Y\ge k$, i.e., Anastrozole treatment increases (39.1%) the chance of higher headaches score compared to Tamoxifen treatment. Similar findings were observed for other B-35 trial items, where of the 28 items analyzed using the same analysis methods, four (14%) reached different conclusions

Figure 1 illustrates the results of each statistical analysis method for the item headache. The hypothesis of equality between the distributions of toxicity scores of tamoxifen and anastrozole groups was not rejected by the PIM Baseline-adjusted TI, Avg, and Max. However, it was rejected (statistically significant difference between arms) for the PIM Baseline as covariate TI, Avg (PI = 0.453, 95%CI 0.419 to 0.488), and Max (PI = 0.456, 95%CI 0.422 to 0.490) statistical analysis methods, indicating a worse (higher) headache score for anastrozole than tamoxifen. Similar results from the approaches with PIM Baseline as covariate were obtained using the CLMM, where anastrozole is associated with a worse (higher) headache score (OR = 1.391, 95%CI 1.040 to 1.861).

As different statistical analysis methods did not lead to the same conclusion, it is not clear whether the differences between arms are true (power = 1–type II error) or false positives (type I error) when the CLMM and PIM baseline as covariate are applied. Inferences should be carried out with the method that provides greatest power to detect differences between treatments, while controlling type I error, therefore, we performed a thorough simulation study to compare the performance of the CLMM and CLMM (Bootstrap) with PIM Baseline-adjusted and PIM Baseline as covariate.

### Simulation study

We performed a simulation study to evaluate the type I and II error rates of CLMM, CLMM (Bootstrap), PIM Baseline-adjusted, and PIM Baseline as covariate analysis methods in evaluating the treatment effects.

The marginal probabilities were set according to the observed proportions of each patient-reported symptom from the B-35 trial and three values for the correlations (*ρ* = 0.2, 0.5, and 0.9) among items over time. These values reflect the range of correlation levels observed in the B-35 trial for different items. We also simulate datasets with different values for the marginal probabilities under the assumption that the treatment groups are different or equal. A total of 270 scenarios were simulated with 225 scenarios showing differences between arms over time. A detailed algorithm on how to generate the datasets is described in Online Supplement Section 5. The R syntax code is available from the corresponding author upon request.

**Fig. 2:**
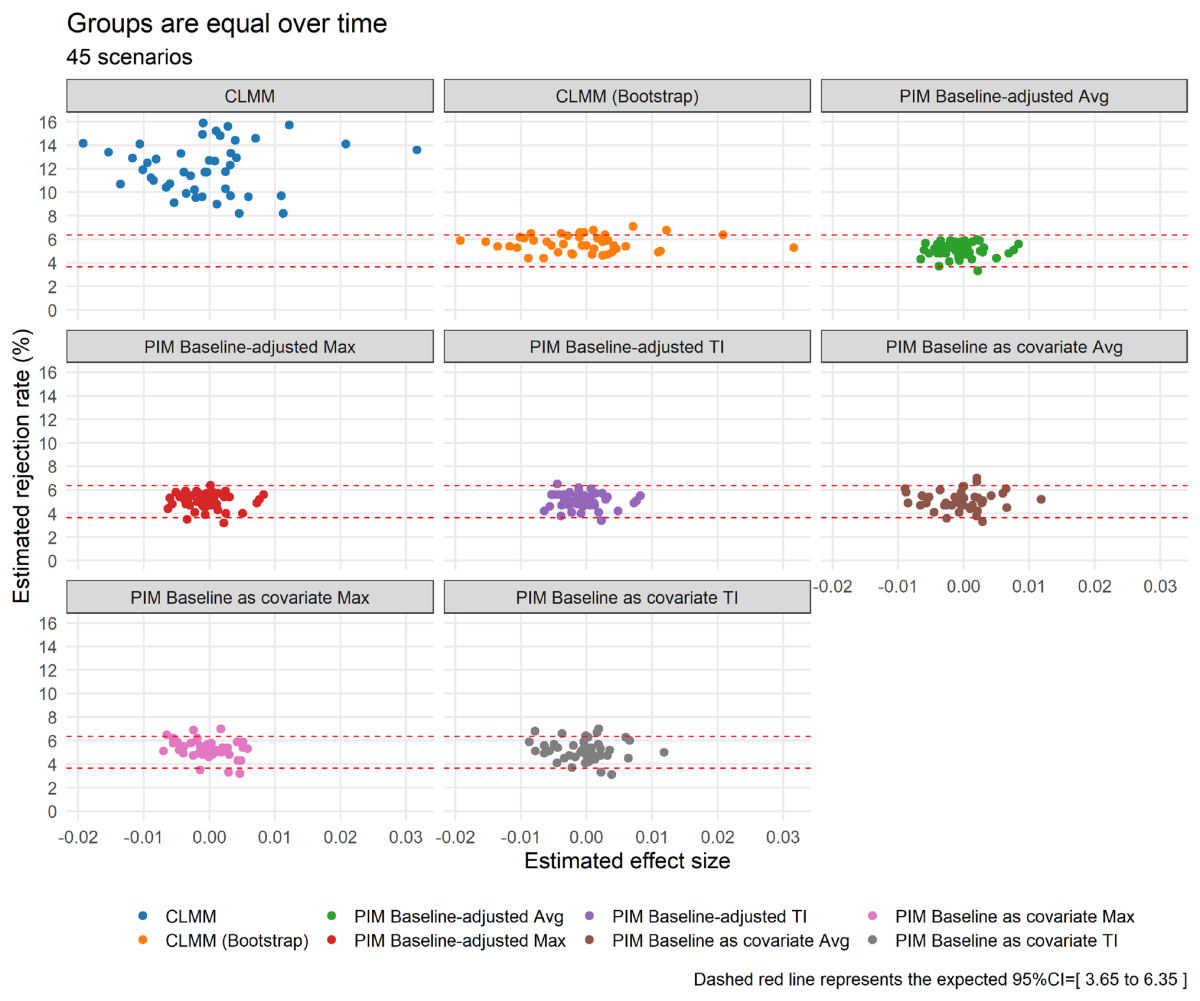
Estimated type I error rates for detecting a significant treatment effect when the groups are equal over time, based on 45 simulated scenarios. Estimated effect size represents the logit of estimated probabilistic index when PIM is used, and under CLMM, it denotes the estimated regression coefficient associated with the group variable

Initially, we evaluate the rejection rates of the null hypothesis when it is true, i.e., no difference between the two arms over time. At a 5% nominal significance level and 1000 trial replicates, we expect the simulated type I error rate to be between 3.65% and 6.35% (95% confidence interval of the fixed nominal level), and any procedure with type I error rate below this range will be considered conservative, and above this range will be considered anti-conservative. The estimated type I error rates based on 45 different scenarios are shown in Fig. 2. For the PIM Baseline-adjusted TI, Avg, Max, and PIM Baseline as covariate TI, Avg, and Max statistical analysis methods, the estimated type I error is close to the nominal level 5%, and in most scenarios, the rates are between 3.65% and 6.35%. On the other hand, the estimated type I error rates associated to the CLMM statistical analysis method are inflated when testing the null hypothesis of treatment effect, yielding minimum type I error rate of 8% (on average it was 12%). These results show that *p* values calculated from usual LRT are somewhat anti-conservative. Further, these *p* values appear to be more anti-conservative for higher correlation among repeated measures. On average, the estimated type I error rates were 10.7%, 11.6%, and 13.9% for correlation values 0.2, 0.5, and 0.9, respectively. However, when the CLMM (Bootstrap) analysis method is applied, it produced smaller type I error rates than CLMM. The empirical rejection rates are reasonably close to the 5% nominal level, regardless of fixed correlation among repeated measures. Overall, the type I error rate was close to 5% (on average it was 5.6%) when CLMM (Bootstrap) was applied, a difference of 6.4% in estimated type I error rate compared to CLMM analysis method. The results suggest that the CLMM (Bootstrap) can produce acceptable type I error rates.

As illustrated in Fig. 2, the statistical analysis methods used in these simulation study produced type I error rates of 5%, except for CLMM. In this case, using the CLMM statistical analysis method may lead to an increase in statistical power because of the inflated type I error rate. Therefore, we considered CLMM (Bootstrap) for fair comparisons.

**Fig. 3:**
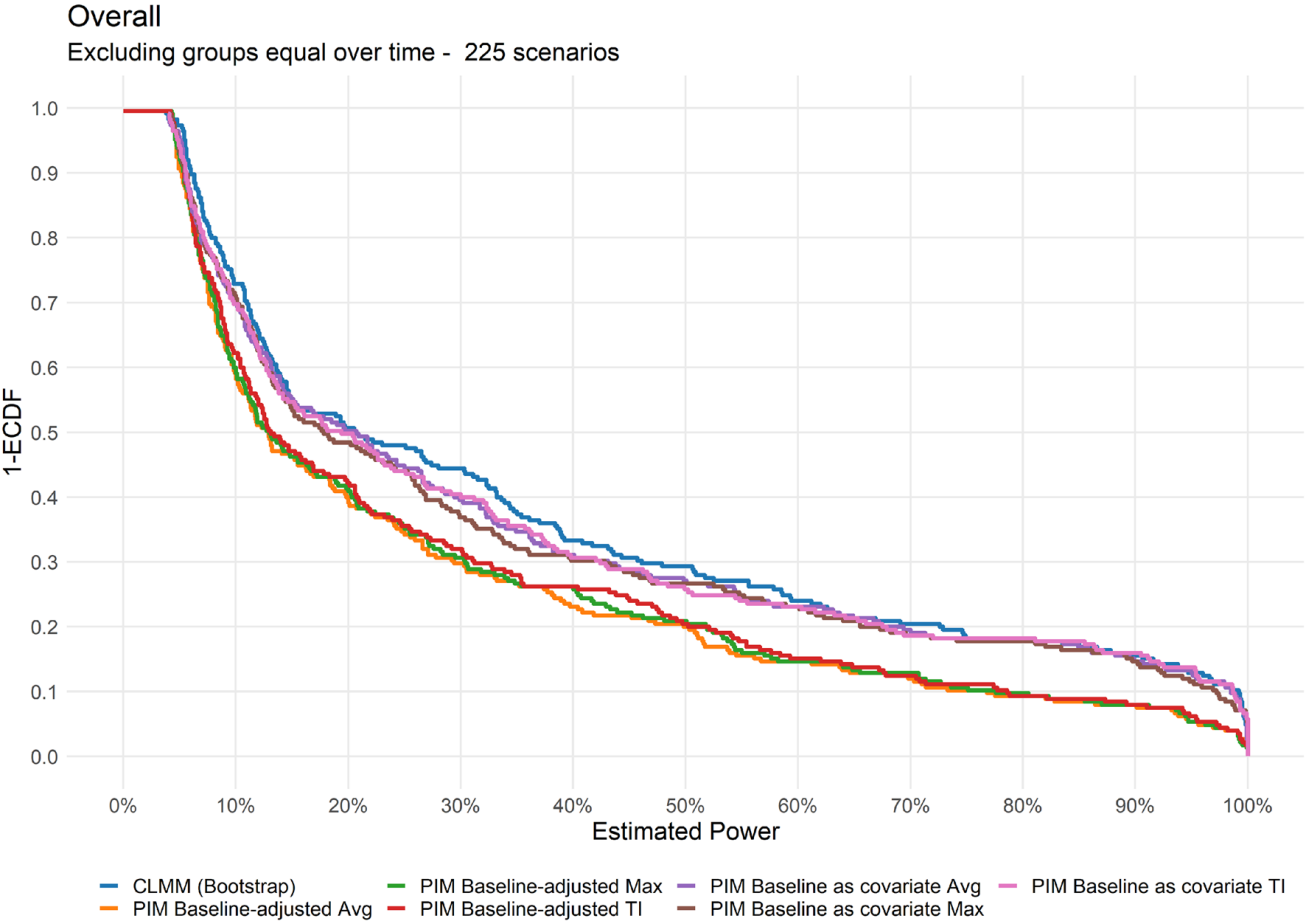
One minus the cumulative distribution function of the overall power estimates by statistical analysis methods under the assumption that the groups are different

Due to the large number of scenarios under study, the power estimates comparisons were performed using a graphical representation and the area under the curve was used to summarize the differences. For each statistical analysis method, the empirical powers were plotted as one minus the empirical cumulative distribution function (1-ECDF). Figure 3 illustrates the power estimates comparisons among the statistical analysis methods, and the area under each curve is shown in Table 2. The y-axis can be interpreted as the probability of a given statistical analysis method to have power greater than a given threshold among 225 scenarios and the x-axis represents possible thresholds based on the power estimates in 225 scenarios. Curves at the top indicate that a larger number of scenarios showed power greater than a given threshold than curves at the bottom.

**Table 2:** Overall power estimates and stratified by fixed correlation among repeated measures by analysis method

Statistical analysis method	Overall*			
	Power^a^	95%CI of power	Mean difference (MD)	95%CI of MD
PIM Baseline-adjusted Max	27.54%	23.80% to 31.28%	8.84%	5.86% to 11.83%
PIM Baseline-adjusted Avg	26.95%	23.25% to 30.65%	9.43%	6.43% to 12.44%
PIM Baseline-adjusted TI	28.14%	24.38% to 31.90%	8.25%	5.32% to 11.17%
PIM Baseline as covariate Max	34.12%	29.78% to 38.46%	2.26%	0.89% to 3.62%
PIM Baseline as covariate Avg	34.90%	30.53% to 39.27%	1.48%	0.90% to 2.08%
PIM Baseline as covariate TI	34.78%	30.41% to 39.15%	1.60%	0.88% to 2.33%
CLMM (Bootstrap)	36.38%	32.01% to 40.75%	Ref	Ref
	*ρ* = 0.2**			
PIM Baseline-adjusted Max	24.54%	18.66% to 30.42%	11.18%	5.3% to 17.06%
PIM Baseline-adjusted Avg	23.72%	17.96% to 29.48%	12.00%	6.08% to 17.92%
PIM Baseline-adjusted TI	25.33%	19.39% to 31.27%	10.40%	4.65% to 16.15%
PIM Baseline as covariate Max	33.40%	26.17% to 40.63%	2.32%	0.31% to 4.35%
PIM Baseline as covariate Avg	34.84%	27.35% to 42.33%	0.88%	0.34% to 1.42%
PIM Baseline as covariate TI	34.76%	27.31% to 42.21%	0.96%	− 0.16% to 2.08%
CLMM (Bootstrap)	35.72%	28.27% to 43.17%	Ref	Ref
	*ρ* = 0.5**			
PIM Baseline-adjusted Max	25.89%	19.65% to 32.13%	8.48%	3.98% to 12.99%
PIM Baseline-adjusted Avg	25.24%	19.08% to 31.40%	9.13%	4.60% to 13.66%
PIM Baseline-adjusted TI	26.42%	20.16% to 32.68%	7.95%	3.55% to 12.33%
PIM Baseline as covariate Max	32.58%	25.09% to 40.07%	1.79%	0.13% to 3.45%
PIM Baseline as covariate Avg	33.38%	25.83% to 40.93%	0.99%	0.39% to 1.60%
PIM Baseline as covariate TI	33.38%	25.77% to 40.99%	0.99%	0.003% to 1.98%
CLMM (Bootstrap)	34.37%	26.90% to 41.84%	Ref	Ref
	*ρ* = 0.9**			
PIM Baseline-adjusted Max	32.19%	24.78% to 39.60%	6.87%	1.63% to 12.1%
PIM Baseline-adjusted Avg	31.88%	24.51% to 39.25%	7.18%	1.90% to 12.4%
PIM Baseline-adjusted TI	32.66%	25.19% to 40.13%	6.40%	1.22% to 11.6%
PIM Baseline as covariate Max	36.39%	28.30% to 44.48%	2.67%	− 0.57% to 5.90%
PIM Baseline as covariate Avg	36.47%	28.48% to 44.46%	2.59%	1.00% to 4.17%
PIM Baseline as covariate TI	36.19%	28.16% to 44.22%	2.87%	1.28% to 4.46%
CLMM (Bootstrap)	39.06%	30.97% to 47.15%	Ref	Ref

^a^Mean power estimates obtained under area 1-ECDF curve of each statistical analysis method. Mean difference (MD) represents the mean difference of power estimates by CLMM compared to other statistical analysis methods. 95%CI was computed using the paired *t* test considering the sample of power estimates. 95%CIs that exclude zero indicate statistically significant differences at the 5% significance level (two-sided hypothesis test)

*Based on 225 simulated scenarios

**Based on 75 simulated scenarios

Our simulations suggest that the CLMM (Bootstrap) statistical analysis method is more powerful than the PIM Baseline-adjusted method to detect difference between treatments (Fig. 3). The power of the PIM Baseline as covariate statistical analysis method was superior to Baseline-adjusted but lower than that of the CLMM (Bootstrap) approach. Specifically, the CLMM (Bootstrap) provided on average higher power estimates (36.38%, 95%CI 32.01% to 40.75%) than PIM Baseline as covariate TI (34.78%, 95%CI 30.41% to 39.15%), Avg (34.9%, 95%CI 30.53% to 39.27%), and Max (34.12%, 95%CI 29.78% to 38.46%) as well as PIM Baseline-adjusted TI (28.14%, 95%CI 24.38% to 31.9%), Avg (26.95%, 23.25% to 30.65%), and Max (27.54%, 95%CI 23.8% to 31.28%).

**Fig. 4:**
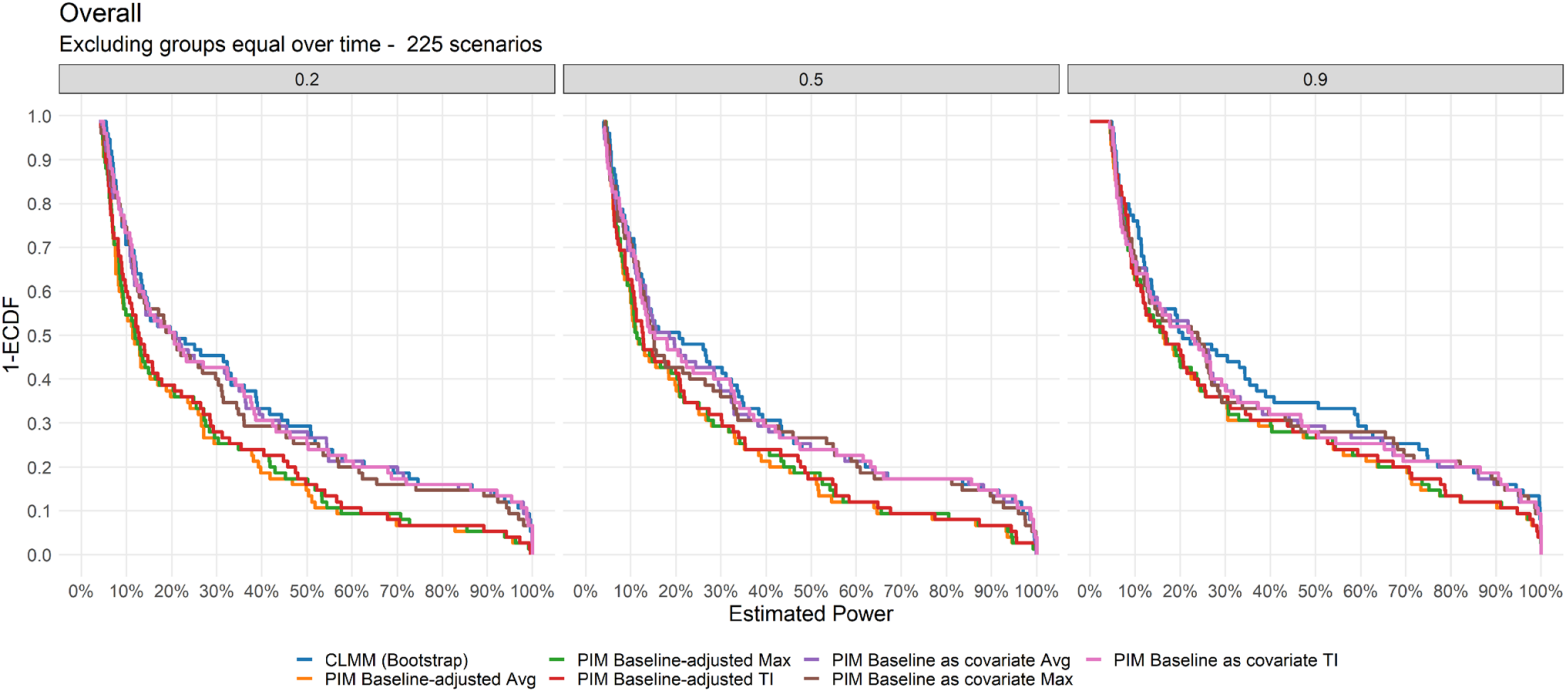
One minus the cumulative distribution function of the overall power estimates by statistical analysis methods stratified by correlations (*ρ* = 0.2, 0.5, and 0.9) among the repeatead measures under the assumption that the groups are different

When comparing statistical methods over scenarios, there was a statistically significant mean difference (MD) of 8.25% (95%CI 5.32% to 11.17%), 9.43% (95%CI 6.43% to 12.44%), and 8.84% (95%CI 5.86% to 11.83%) between the power estimates by CLMM (Bootstrap) analysis method and PIM Baseline-adjusted TI, Avg, and Max, respectively. A statistically significant MD was also observed between the power estimates by CLMM (Bootstrap) versus PIM Baseline as covariate TI (MD = 1.6%, 95%CI 0.88% to 2.33%), Avg (MD = 1.48%, 95%CI 0.9% to 2.08%), and Max (MD = 2.26%, 95%CI 0.89% to 3.62%). The same pattern was observed in the subset of simulations with low (*ρ* = 0.2), moderate (*ρ* = 0.5), and high (*ρ* = 0.9) correlation among the repeated measures, where higher MDs between CLMM (Bootstrap) and PIM Baseline-adjusted, and greater than PIM Baseline as covariate (Table 2 and Fig. 4).

When comparing the results excluding the CLMM statistical method, there was a statistically significant MD of 6.64% (95%CI 3.81% to 9.47%), 7.95% (95%CI 4.95% to 10.94%), and 6.58% (95%CI 3.97% to 9.2%) between the power estimates by PIM Baseline as covariate TI, Avg, and Max and PIM Baseline-adjusted TI, Avg, and Max, respectively. The same pattern was also observed in the subset of simulations with low and moderate correlation among the measurements. There were no differences between the power estimates by PIM Baseline as covariate and PIM Baseline-adjusted under the assumption of high correlation among the repeated measures (Online Supplement Section 5, Table S5).

Similar results were also observed within each simulated data type (Online Supplement Section 1 describes all simulated scenarios). Higher MDs of power estimates occurred under the assumption that the groups are equal only at baseline and different at times 2 and 3. A statistically significant MD ranging from 16.51% to 17.93% was observed between CLMM (Bootstrap) and PIM Baseline-adjusted analysis methods, and from 2.81% to 3.98% between CLMM (Bootstrap) and PIM Baseline as covariate. When the simulated data followed the assumptions that the groups are different at baseline with same increments in times 2 and 3, a statistically significant MD of 5.37% to 5.78% and 1.75% to 2.33% was observed between CLMM (Bootstrap) and PIM Baseline-adjusted and between the CLMM (Bootstrap) and PIM Baseline as covariate, respectively. Under the B-35 trial scenarios, a statistically significant MD ranging from 5.41% to 6.72% occurred between the CLMM (Bootstrap) and PIM Baseline-adjusted, while a MD varying of 1.52% to 2.60% was observed between CLMM (Bootstrap) and PIM Baseline as covariate. There were no differences in power estimates among the methods under the assumption that the groups are different in all time points (see the Online Supplement Section 5, Table S4). When excluding the CLMM statistical analysis method, higher MDs of power estimates occurred between PIM Baseline as covariate and PIM Baseline-adjusted under the assumption that the groups are equal only at baseline and different at times 2 and 3. There was a statistically significant MD of 13.7% (95%CI 6.7% to 20.69%), 15.06% (95%CI 7.8% to 22.32%), and 13.01% (95%CI 6.44% to 19.59%) between the power estimates by PIM Baseline as covariate TI, Avg, and Max and PIM Baseline-adjusted TI, Avg, and Max, respectively. Under the assumptions that the groups are different at baseline with same increments in times 2 and 3, a statistically significant MD of 4.46% (95%CI 0.33% to 8.59%) and 3.45% (95%CI 0.2% to 6.7%) was observed between PIM Baseline as covariate Avg, and Max and PIM Baseline-adjusted Avg, and Max, respectively. When the simulated data followed the B-35 trial scenarios, a statistically significant MD of 3.89% (95%CI 2.49% to 5.29%), 5.36% (95%CI 3.53% to 7.18%), and 3.51% (95%CI 2.21% to 4.82%) occurred between PIM Baseline as covariate TI, Avg, Max and PIM Baseline-adjusted TI, Avg, Max, respectively. Under the assumption that the groups are different in all time points, there were no differences in power estimates between PIM Baseline as covariate and PIM Baseline-adjusted statistical analysis methods (see the Online Supplement Section 5, Table S5).

## Discussion

Patient-reported symptom items (e.g., PRO-CTCAE) are increasingly considered as indicators of toxicity in [16, 37–40] clinical trials. Different from clinician-reported CTCAE data, PRO-CTCAE items are collected from patients at baseline to measure symptom burden before treatment, allowing for separation of burden caused from disease and treatment. While overall summary scores are often used to evaluate toxicity, single items provide information about specific adverse events. Therefore, robust statistical methods are needed for single items that incorporate baseline symptoms while capturing the overall patients’ toxicity burden over time. This paper provides a comprehensive simulation study comparing analytical methods for single-item longitudinal PRO data from the B-35 clinical trial.

Our simulations indicate that the CLMM had an estimated type I error rates routinely higher than the 5% nominal significance level. In addition, higher estimated type I error rates were observed as the correlation among the repeated measures increased. In contrast, the LRT parametric bootstrap (CLMM (Bootstrap)) was able to produce acceptable type I error rates. Similar results close to 5% were also observed for the PIM Baseline-adjusted and PIM Baseline as covariate analysis methods.

Simulation results revealed important differences in power estimates among the analysis methods that maintained the type I error rate close to 5% (i.e., excluding CLMM): CLMM (Bootstrap) analysis method provided substantially higher power estimates than PIM Baseline-adjusted and a smaller but significant improvement in power compared to PIM Baseline as covariate. There was a statistically significant MD of approximately 9% between the power estimates of CLMM (Bootstrap) and PIM Baseline-adjusted analysis methods; and a statistically significant MD of roughly 2% between CLMM (Bootstrap) and PIM Baseline as covariate analysis methods. Among the PIM statistical methods, higher power estimates were observed for the PIM Baseline as covariate with a statistically significant MD of approximately 6.5% between the power estimates of PIM Baseline as covariate Max, TI and PIM Baseline-adjusted Max, TI; and a statistically significant MD about 8% between PIM Baseline as covariate TI and PIM Baseline as covariate TI. The same pattern was also observed for the simulated scenario that mimicked the observed proportions of B-35 trial items and the other different scenarios, except when the groups were different in all time points. Therefore, the difference between tamoxifen and anastrozole for headache detected by CLMM and PIM Baseline as covariate is plausible given both methods showed greater power than PIM Baseline-adjusted analysis method.

Although the PIM Baseline as covariate is a semiparametric approach, it provided average power close to the CLMM (Bootstrap) analysis method in most of the simulated scenarios. The small difference between average powers obtained by both analysis methods may be associated with the small number of assessment time points after baseline (times 2 and 3). The CLMM analysis method considers all patient's follow-up scores when modeling, while PIM Baseline as covariate summarizes them into a single value. With a greater number of assessments during follow-up, the difference between analytical methods might increase. Another study is needed to evaluate the performance of the analysis methods for a greater number of assessments. The current study found, consistent with previous work [17], that the PIM Baseline-adjusted analysis method shows lower power.

Several strengths are associated with the use of CLMM. It considers all the patient's experience over the course of the trial by accounting for the dependency structure among ordinal repeated measures, without losing interpretability. This additional information provides, on average, greater power to detect differences between treatments compared to the PIM Baseline-adjusted and PIM Baseline as covariate analysis methods, resulting in fewer number of patients needed to detect treatment differences. However, it shows unacceptable type I error rate without bootstrap, yielding minimum type I error rate of 8% (on average it was 12%), and when LRT by parametric bootstrap is applied, it is computationally intensive. In addition, it relies on assumptions of proportional odds, normality of the random effects and that the data generator process in the parametric bootstrap is the same as the data generator process of the observed data. On the other hand, PIM Baseline as covariate can provide results close to the CLMM (Bootstrap) method when the number of assessments is small, which is an advantage, because the PIM does not require any distribution assumption and it is computationally fast.

The limitations of this study are that these methods were applied in simulated datasets of 100 patients per group measured in three time points with same correlation among them (0.2, 0.5, and 0.9), and therefore, the impact of different sample sizes and/or repeated measures on type I error rates and power were not evaluated. In addition, the sample generator was based on copula multinomial model, an approach different from those considered in this study, which made it possible to generate datasets according to the item severities in both groups, and to vary correlations among the measures. The results for CLMM are based on simulations using random intercept mixed model with three fixed effects, so the observed type I error rates might not hold up for a model with a more complex structure and smaller sample sizes.

As the performance of the semiparametric approaches with baseline as covariate is close to the parametric approach, we hypothesize that parametric approaches using TI or other summary measures may be even more powerful methods to analyze longitudinal data with single items. Nevertheless, the quasi-continuous nature [41] of TI currently constrains the use of common parametric models. Further research is necessary, but the results of this study allow us to feel relatively confident that anastrozole treatment was associated with worse headache symptoms.

## Conclusion

The CLMM showed unacceptable type I error rate without bootstrap, while the CLMM with bootstrap provided the best performance among analytical methods. However, it relies on assumptions difficult to verify and that might not be fulfilled in the real world, and therefore, our recommendation is the use of PIM models using Max or TI score with baseline as covariate considering the lack of interpretability of average AE score discussed in our previous work [41].

## Supplementary Information

Below is the link to the electronic supplementary material.

Supplementary file1 (DOCX 1011 KB)

## Data Availability

The data that support the findings in the case study are available from NRG Oncology but restrictions apply to the availability of these data, which were used under license for the current study, and so are not publicly available. Requests for the data, however, can be made to NRG Oncology at https://www.nrgoncology.org/Resources/Ancillary-Projects-Data-Sharing-Application.
