# Supplementary material for: Simulation study comparing analytical methods for single-item longitudinal patient-reported outcomes data
Source: Qual Life Res. 2022 Oct 17;32(3):827–39. doi: 10.1007/s11136-022-03267-z (PMC9992042; doi:10.1007/s11136-022-03267-z)
Supplement: Supplementary file 1 — Supplementary file1 (DOCX 1011 KB) [file 11136_2022_3267_MOESM1_ESM.docx]

**Supplementary material**

**Article:** Simulation Study Comparing Analytical Methods for Single Item Longitudinal Patient-Reported Outcomes Data

**Journal:** Quality of Life Research

**Authors:** Vinicius F. Calsavara, PhD^1*^; Márcio A. Diniz, PhD^1^; Mourad Tighiouart, PhD^1^; Patricia A. Ganz, MD^2^; N. Lynn Henry, MD, PhD^3^; Ron D. Hays, PhD^4^; Greg Yothers, PhD^5^; André Rogatko, PhD^1^

1 Samuel Oschin Comprehensive Cancer Institute, Cedars-Sinai Medical Center, Los Angeles, CA, USA

2 University of California Los Angeles Jonsson Comprehensive Cancer Center, Los Angeles, CA, USA

3 University of Michigan Rogel Cancer Center, Ann Arbor, MI, USA

4 University of California Los Angeles, Department of Medicine, Los Angeles, CA, USA

5 University of Pittsburgh, Pittsburgh, PA, USA

**Caption:**

This online resource provides a detailed description of simulated scenarios for single item longitudinal data; methods for summarizing the toxicity in a single measure; a briefly description of the cumulative logit mixed model to analyze single item longitudinal ordinal data; an algorithm to generate the datasets and additional results of simulation study.

Section 1) Fixed proportions of the categories in each time point by group to generate the datasets in the simulation study according to the five simulated data types and a description of simulated scenarios for single item longitudinal data given in Table S1; Figures 1-2 show the observed proportions of each item and arm of B-35 trial used to generate datasets. Figures 3-6 show the arbitrary values for the marginal probabilities under the assumption that the groups are: i) Different in all time points; ii) Equal only at baseline, different at times 2 and 3; iii) Different at baseline with same increments at times 2 and 3; and iv) Equal over time. Section 2) The mathematical formulation of baseline-adjusted and post-baseline methods to summarize the AE over time in a single measure with an example of this calculation (Tables S2-S3); Section 3) The cumulative logit mixed model and an algorithm to conduct the likelihood ratio test by parametric bootstrap; Section 4) The violin plot of headache score from the B-35 trial; Section 5) A detailed algorithm to generate the dataset and performing the simulation study; Additional results of the simulation studies. Figure 11 presents the comparisons of empirical powers (1-ECDF) of the statistical analysis methods within each simulated data type. The mean power estimates of each statistical analysis method and the mean difference (MD) of power estimates by the parametric cumulative logit mixed model (CLMM) compared to the probabilistic index model (PIM) baseline-adjusted and baseline as covariate is shown in Table S4. In addition, the MD of power estimates by the PIM Baseline as covariate compared to the PIM Baseline-adjusted is shown in Table S5.


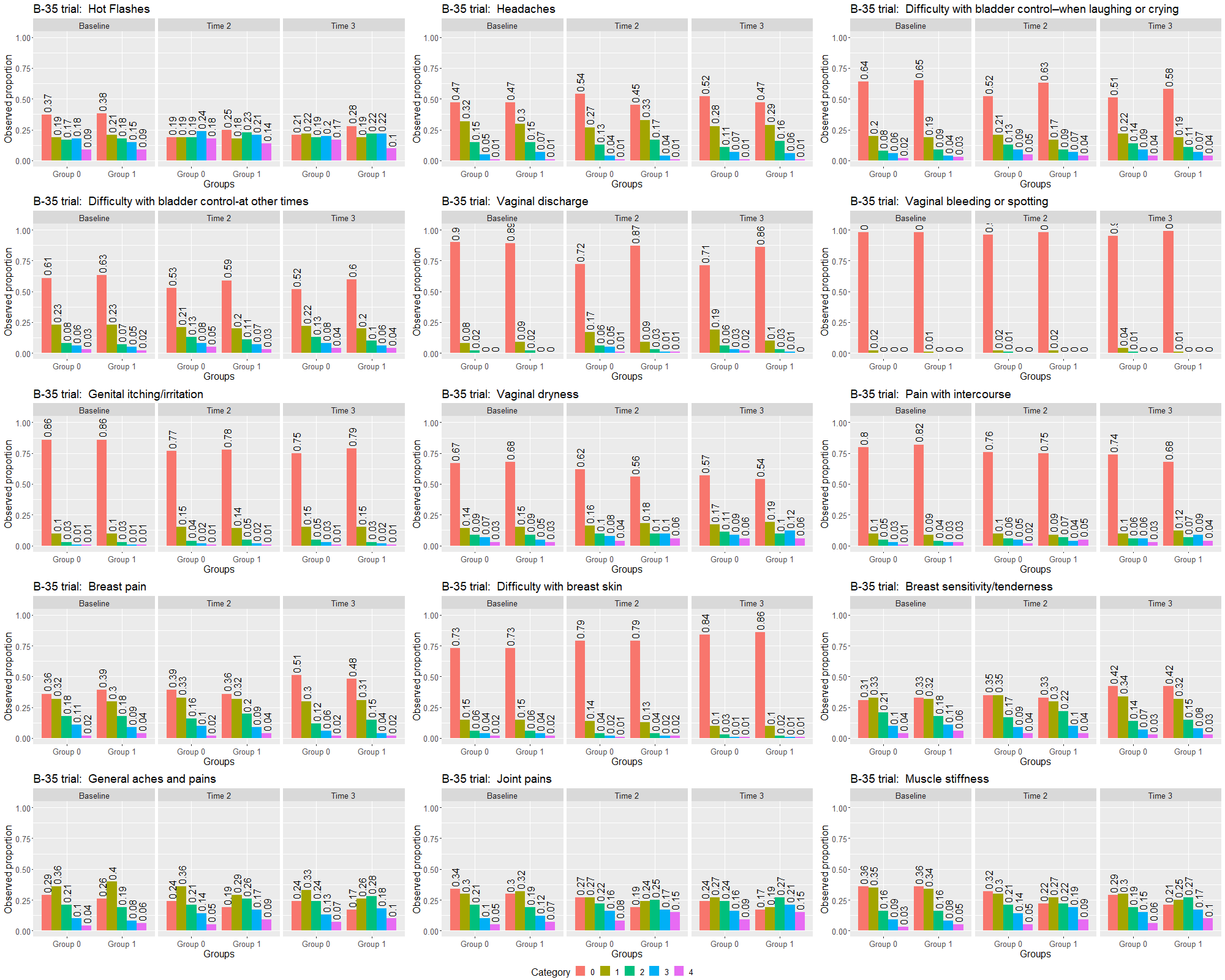
**1 Observed and fixed proportions for each category item**

**Fig. 1** Observed proportions of each category item by time point and treatment group of B-35 trial. Tamoxifen and Anastrozole are denoted by groups 0 and 1, respectively.


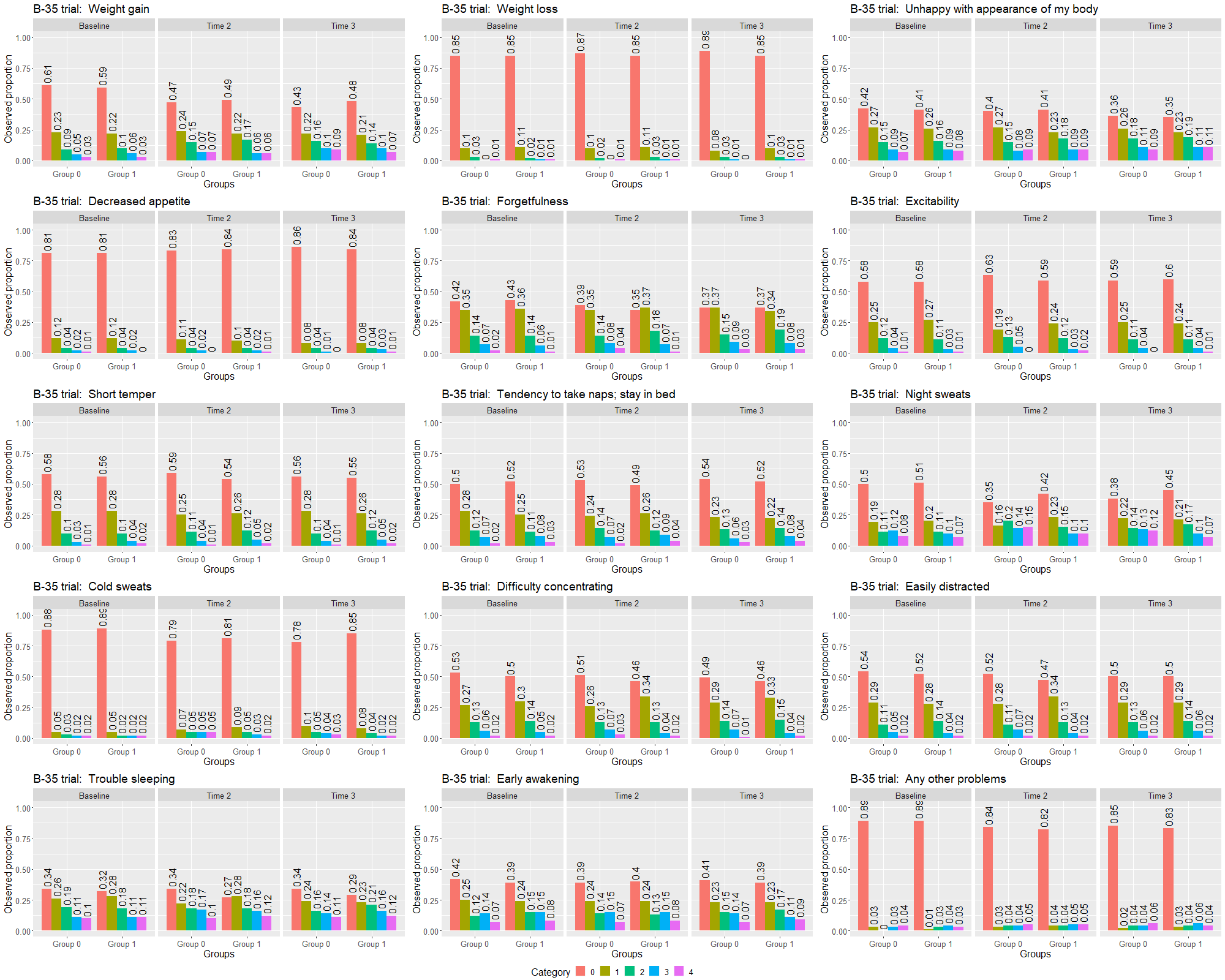
**Fig. 2** Observed proportions of each category item by time point and treatment group of B-35 trial. Tamoxifen and Anastrozole are denoted by groups 0 and 1, respectively.


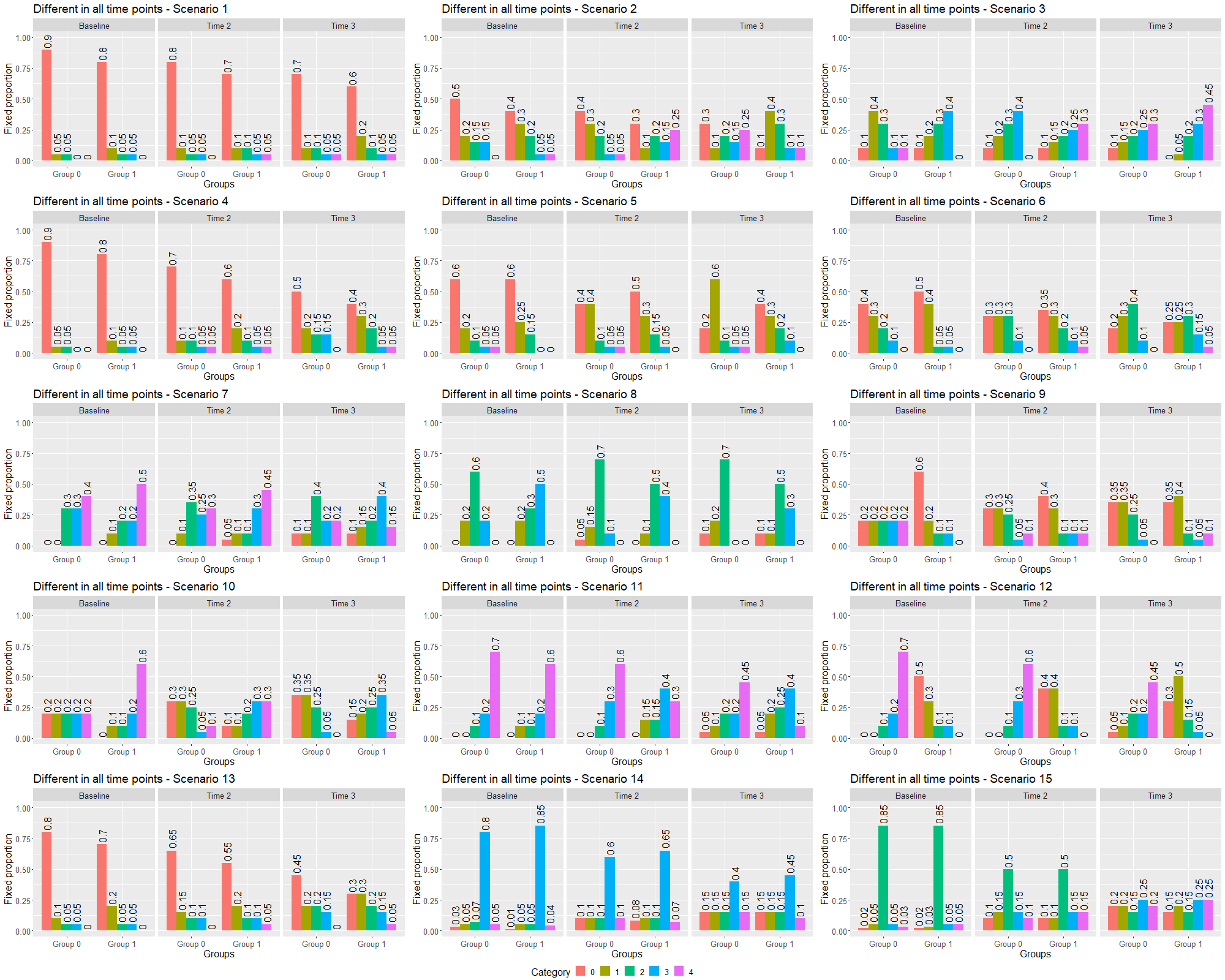
**Fig. 3** Fixed proportions in each category for a single item longitudinal data under the assumption the groups are different in all time points.


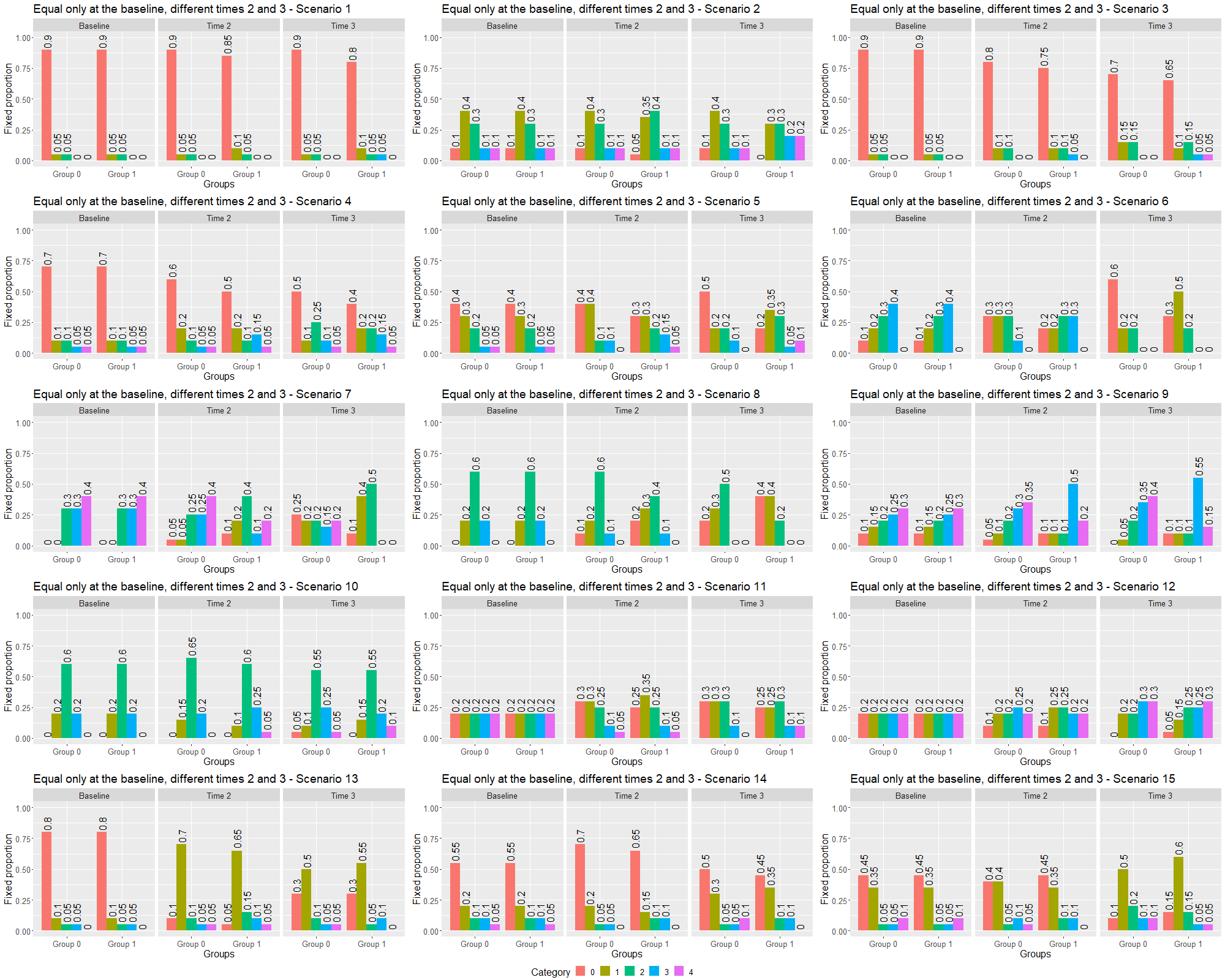
**Fig. 4** Fixed proportions in each category for a single item longitudinal data under the assumption the groups are equal only at baseline and different at times 2 and 3.


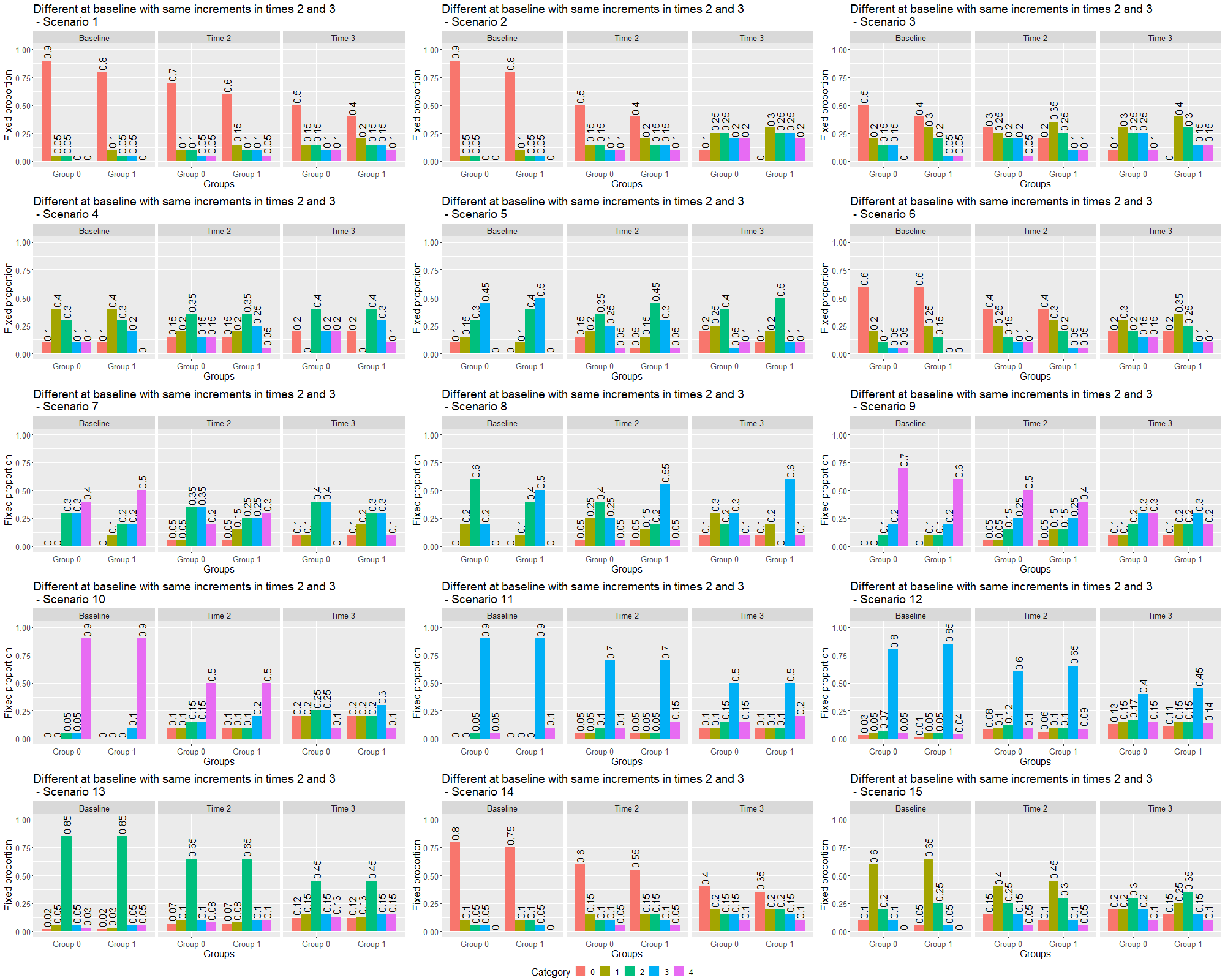
**Fig. 5** Fixed proportions in each category for a single item longitudinal data under the assumption of the groups are different at baseline with the same increments at times 2 and 3.


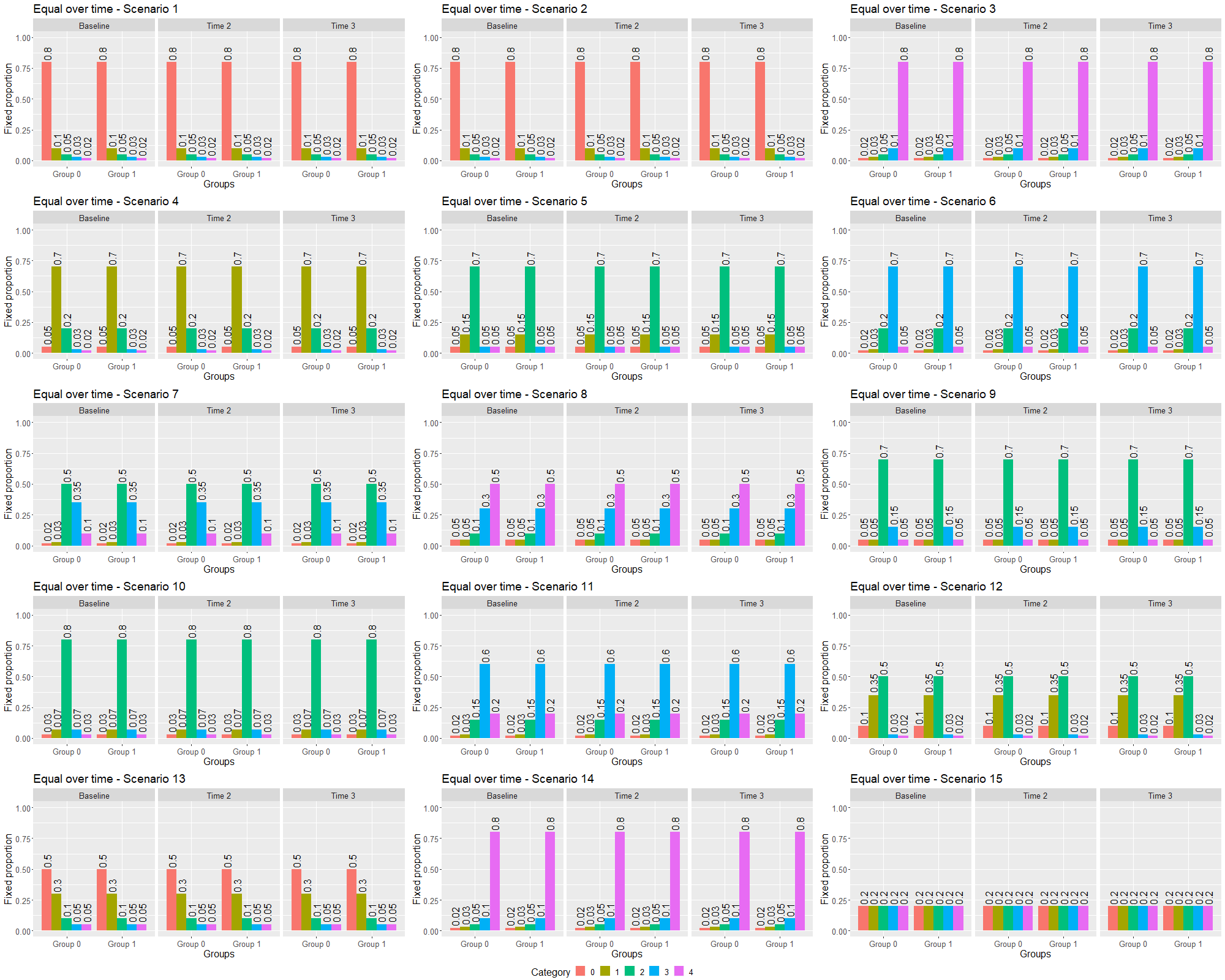
**Fig. 6** Fixed proportions in each category for a single item longitudinal data under the assumption the groups are the same over time.

**Description of simulated scenarios**

**Table S1** Description of 270 simulated scenarios for single item longitudinal data

| **Simulated data type** | **Description of generated dataset** | **Groups are equal in the generated dataset** | **Number of simulated scenarios for each fixed correlation (ρ=0.2, 0.5, 0.9)** | **Number of simulated scenarios*** | **Fixed proportions used to generate the datasets are reported** |
| --- | --- | --- | --- | --- | --- |
| B-35 trial | Using observed proportions of B-35 trial items by group over three time points | No | 30 | 90 | Figures 1-2 |
|  |  |  |  |  |  |
| Different in all time points | Different values of the marginal probabilities (MPs) were fixed in each group over time points | No | 15 | 45 | Figure. 3 |
|  |  |  |  |  |  |
| Equal only at baseline, different times 2 and 3 | At baseline, the same values of the MPs were fixed in both groups, while at times 2 and 3 different values of MPs were considered in each group | No | 15 | 45 | Figure. 4 |
|  |  |  |  |  |  |
| Different at baseline with same increments in times 2 and 3 | At baseline, different values of the MPs were fixed in each group, while at times 2 and 3 the MPs were defined as increments of baseline MPs. | No | 15 | 45 | Figure. 5 |
|  |  |  |  |  |  |
| Equal over time | The same values of the MP were fixed in both groups over time | Yes | 15 | 45 | Figure. 6 |

Marginal probabilities (MPs) denote the fixed probabilities of each category (graded from 0 to 4), such that the sum is one, to generate the datasets. *1000 different datasets were generated for each scenario to estimate the type I and II error rates.

**2 Mathematical definitions**

**Methods for summarizing toxicity in a single measure**

To compute the toxicity index for a given individual patient, their observed scores over time are first sorted in descending order ${(X}_{1}\geq X_{2}\geq\ldots{\geq X}_{J})$, and then the statistic is calculated as follows

$$\mathrm{TI}=\sum_{i=1}^{J} \frac{X_{i}}{\prod_{l<i} (1+X_{l})},$$

where $J$ denotes the number of measurements observed over time. The resulting toxicity index statistic quantifies the toxicities experienced by an individual patient over the trial period.

**Methods for analyzing toxicity using pre-existing symptoms at baseline in a single item longitudinal data**

**Post-baseline**

Assume that we have a single item longitudinal data measured in $J$ time points $(J>1)$. In each assessment, the item score can assume any value on discrete interval${[K]}_{*}=\{0, 1, \ldots, K\}$. For each subject, we observe de item scores that can be viewed as a vector ${\boldsymbol{X}=(X}_{0}, X_{1}, \ldots, X_{J-1})\in{[K]}_{*}^{J}$, where $X_{i}\in{[K]}_{*}$ denotes the item score in time $i$. The time zero represents the baseline, while times greater than zero denote the follow-up time point. We define the vector $\boldsymbol{X}^{0}=\left( X_{1},X_{2},\ldots, X_{J-1} \right)$ using only follow-up scores. Let $X_{(1)}\geq X_{\left( 2 \right)}\geq\ldots\geq X_{(J-1)}$ be the order statistic of $\boldsymbol{X}^{0}$.

The post-baseline method to summarize the item scores $\boldsymbol{X}$ in a single value is obtained using only follow-up scores and several summary measures can be applied. The following three endpoints are of interest:

The post-baseline TI (PBTI) is computed as following,

$$\mathrm{PBTI} \left( \boldsymbol{X}^{0} \right)=\sum_{i=1}^{j-1} \frac{X_{\left( i \right)}}{w_{i}(\boldsymbol{X}^{0})}, \mathrm{where} w_{i}\left( \boldsymbol{X}^{0} \right)=\prod_{l=1}^{i-1} (1+X_{\left( l \right)}).$$

The post-baseline average (PBAvg) is given by

$$\mathrm{PBAvg} \left( \boldsymbol{X}^{0} \right)=\frac{\sum_{i=1}^{J-1} X_{\left( i \right)}}{J-1}.$$

The post-baseline max (PBMax) is

$$\mathrm{PBMax} \left( \boldsymbol{X}^{0} \right)=X_{(1)}.$$

The probabilistic index model (PIM) was fitted to compare the distributions of each endpoint between arms considering the baseline measurement ${(X}_{0})$as covariate. These analysis methods were denoted throughout in article as PIM baseline as covariate TI, Avg, and Max depending on summary measure used.

**Baseline-adjusted**

To summarize the vector ${\boldsymbol{X}=(X}_{0}, X_{1}, \ldots, X_{J-1})\in{[K]}_{*}^{J}$ in a single value using baseline-adjusted method, we define the vector $\boldsymbol{Y=}(Y_{1}, Y_{2},\ldots, Y_{J-1})$, where $Y_{i}=X_{i}I(X_{i}>X_{0})$ with $I(X_{i}>X_{0})$ represents an indicator variable, i.e., 1 if $X_{i}>X_{0}$, otherwise is zero. In addition, let $\boldsymbol{Y}_{\boldsymbol{+}}\boldsymbol{=Y-}0$ the reduced vector of dimension $W$ that retains the nonzero scores and $Y_{(1)}\geq Y_{\left( 2 \right)}\geq\ldots\geq Y_{(W)}$ the order statistic of $\boldsymbol{Y}_{\boldsymbol{+}}$. The baseline-adjusted method is computed using reduced vector $\boldsymbol{Y}_{\boldsymbol{+}}$ as following:

The baseline-adjusted TI (BATI) is given by

$$\mathrm{BATI} \left( \boldsymbol{X} \right)=\sum_{i=1}^{W} \frac{Y_{\left( i \right)}}{w_{i}(\boldsymbol{Y}_{+})}, \mathrm{where} w_{i}\left( \boldsymbol{Y}_{+} \right)=\prod_{l=1}^{i-1} (1+Y_{\left( l \right)}).$$

The baseline-adjusted average (BAAvg) can be represented as

$$\mathrm{BAAvg} \left( \boldsymbol{X} \right)=\frac{\sum_{i=1}^{W} Y_{\left( i \right)}}{W}.$$

The baseline-adjusted max (BAMax) is

$\mathrm{BAMax} \left( \boldsymbol{X} \right)=Y_{(1)}$.

Baseline-adjusted is zero if the vector $\boldsymbol{Y}_{\boldsymbol{+}}$ is empty, regardless of summary measure applied.

The PIM was fitted to compare the distributions of each endpoint between arms. This statistical analysis method was denoted throughout in article as Baseline-adjusted TI, Avg, and Max depending on summary measure applied.

Tables S2-S3 provide an example of calculation of toxicity index (TI), average, maximum using the baseline-adjusted and post-baseline methods for a single item longitudinal data of a given patient.

**Table S2** Example of TI, Avg, and Max using patient reported outcome adverse event score over time for a given patient

| Time point | Score |  |  | Ordered descending score* | Time rank | Accrued toxicity |
| --- | --- | --- | --- | --- | --- | --- |
| Baseline | 2 |  |  | 4 | Time 5 | 4 |
| Time 2 | 3 |  |  | 3 | Time 2 | +0.6 |
| Time 3 | 1 |  |  | 3 | Time 6 | +0.15 |
| Time 4 | 1 |  |  | 2 | Baseline | +0.025 |
| Time 5 | 4 |  |  | 1 | Time 3 | +0.004 |
| Time 6 | 3 |  |  | 1 | Time 4 | +0.002 |
|  |  |  |  |  |  | 4.781 |
| Summary measure | Value |  |  |  |  |  |
| TI | 4.781 |  |  |  |  |  |
| Avg | 2.333 |  |  |  |  |  |
| Max | 4 |  |  |  |  |  |

*Adverse event scores are show in ordered descending in severity with respective time point provided in Time rank column.

**Table S3** Example of baseline-adjusted and post-baseline TI, Avg, and Max using patient reported outcome adverse event score over time for a given patient

| Time points | Score | Worse than baseline |  | Baseline-adjusted | Post-baseline |
| --- | --- | --- | --- | --- | --- |
|  |  |  |  | Selected score | Selected score |
| Baseline | 2 | - |  | - | - |
| Time 2 | 3 | Yes |  | 3 | 3 |
| Time 3 | 1 | No |  | - | 1 |
| Time 4 | 1 | No |  | - | 1 |
| Time 5 | 4 | Yes |  | 4 | 4 |
| Time 6 | 3 | Yes |  | 3 | 3 |
|  |  |  |  |  |  |
| Summary measure |  |  |  | Value | Value |
| TI |  |  |  | 4.75 | 4.769 |
| Avg |  |  |  | 3.33 | 2.4 |
| Max |  |  |  | 4 | 4 |

Baseline-adjusted TI, Avg, and Max were computed using only the scores than were worse in severity than baseline provided in selected score column. Post-baseline TI, Avg, and Max were computed using only the scores in follow-up.

The treatment arms were compared using the baseline-adjusted score distributions through of probabilistic index model (PIM). We also considered the post-baseline score distributions using the baseline score as covariate in PIM, denoted in article as baseline as covariate statistical analysis method.

**3 Analysis methods for single item longitudinal ordinal data**

**Cumulative logit mixed model**

Let us denote by $Y_{i}$ a random variable representing the AE score that takes a value $k$ if the $i$th ordinal observations falls in the $k$’th category, where $k=0, \ldots, K-1$ and $K\geq2$. The cumulative logit mixed model is given by

$$\log\left\{ \frac{P\left( Y_{i}\leq k \right)}{1-P\left( Y_{i}\leq k \right)} \right\}=\theta_{k}-\boldsymbol{X}_{i}\boldsymbol{\beta}-b_{i},$$

where $\theta_{k}$ is the intercept (threshold parameter) of the $k$’th category (such as $\theta_{0}<\theta_{1}<\ldots< \theta_{K-1}$), $\boldsymbol{X}_{i}$ is a $n\times p$ matrix of the predictor variables, $\boldsymbol{\beta}$ is a $p\times1$ column vector of the fixed-effects regression coefficients, and $b_{i}$ is the random subject effect, which indicates the influence of patient $i$ on his/her repeated assessment. Usually, the distribution of a random effect is assumed to be a normal distribution with zero mean and variance $\sigma_{b}^{2}$.

If $\boldsymbol{X}$ represents a group variable with two levels (0: control and 1: intervention), then the odds ratio (OR) of the event $Y_{i}\leq k$ is $exp(-\beta_{\mathrm{intervention}})$. Likewise, the OR of the event $Y_{i}>k$ is $exp(\beta_{\mathrm{intervention}})$. This assumption states that $\beta$ is independent of the level $k$, or that the effect of $X$ is the same for all levels $k$ of the ordinal response.

Commonly, the likelihood ratio test (LRT) is used to make inference on fixed and random effects. It compares two different models (unconstrained and constrained models) to evaluate whether one is better fit to the data than the other. It is applied to decide if a particular random effect (e.g., random subject effect) or fixed effect (e.g., treatment effect) should be included in the model by evaluating whether that effect improves the fit of the model, with all other model parameters held constant [1]. The p-value associated with the LRT is obtained using a chi-square distribution with degrees of freedom equal to the difference in number of parameters between unconstrained and constrained models.

The parametric bootstrap procedure generates pseudo-samples from the constrained model (under the null hypothesis) and then fits both unconstrained and constrained models to the generated samples, which produces an empirical distribution of the LR under the null hypothesis. The bootstrap p-value corresponds to the percentage of simulated LR values that are larger than the LR statistic value obtained in the original sample.

**Likelihood ratio test by parametric bootstrap**

**Algorithm.** For a given sample size in each group define:

1. Number of measures over time;
2. For each group and time point, set the expected proportion in each category of the response variable (with five categories), such their sum is one;
3. The desired correlation value among the repeated measures;
4. With the fixed parameter values generate the random sample of the multinomial copula model;
5. Fit the cumulative logit link model (CLMM) considering the measures in time points 2 and 3 as response variable and the baseline measurement and group as covariates (fixed effects);
6. Fit the model CLMM of step 5 without group covariate;
7. Computed the likelihood ratio test (LRT);

**Parametric bootstrap sampling - under null hypothesis (no difference between the groups)**

1. Fit the multinomial copula model to the generated dataset (step 4) assuming that there is not a difference between the two groups;
2. Generate the random sample from multinomial copula model replacing the parameters by their estimates obtained in step I.
3. Randomly split the generated dataset into the same proportion of patients from groups 0 and 1.
4. Fit the CLMM considering the measures in time points 2 and 3 as response variable and the baseline measurement and group as covariates (fixed effects) in generated data in step III;
5. Fit the CLMM of step IV without group covariate;
6. Computed the LRT;
7. Repeat the steps II-VI according to the number (B) of bootstrap sampling desired.
8. Compute the bootstrap p-value as following:
   1. Count the number of LRT values (obtained in Step VI) that are greater than or equal to the observed LRT value (computed in Step 7) and divided it by B.

**4 Violin plot of headache score from the B-35 trial**


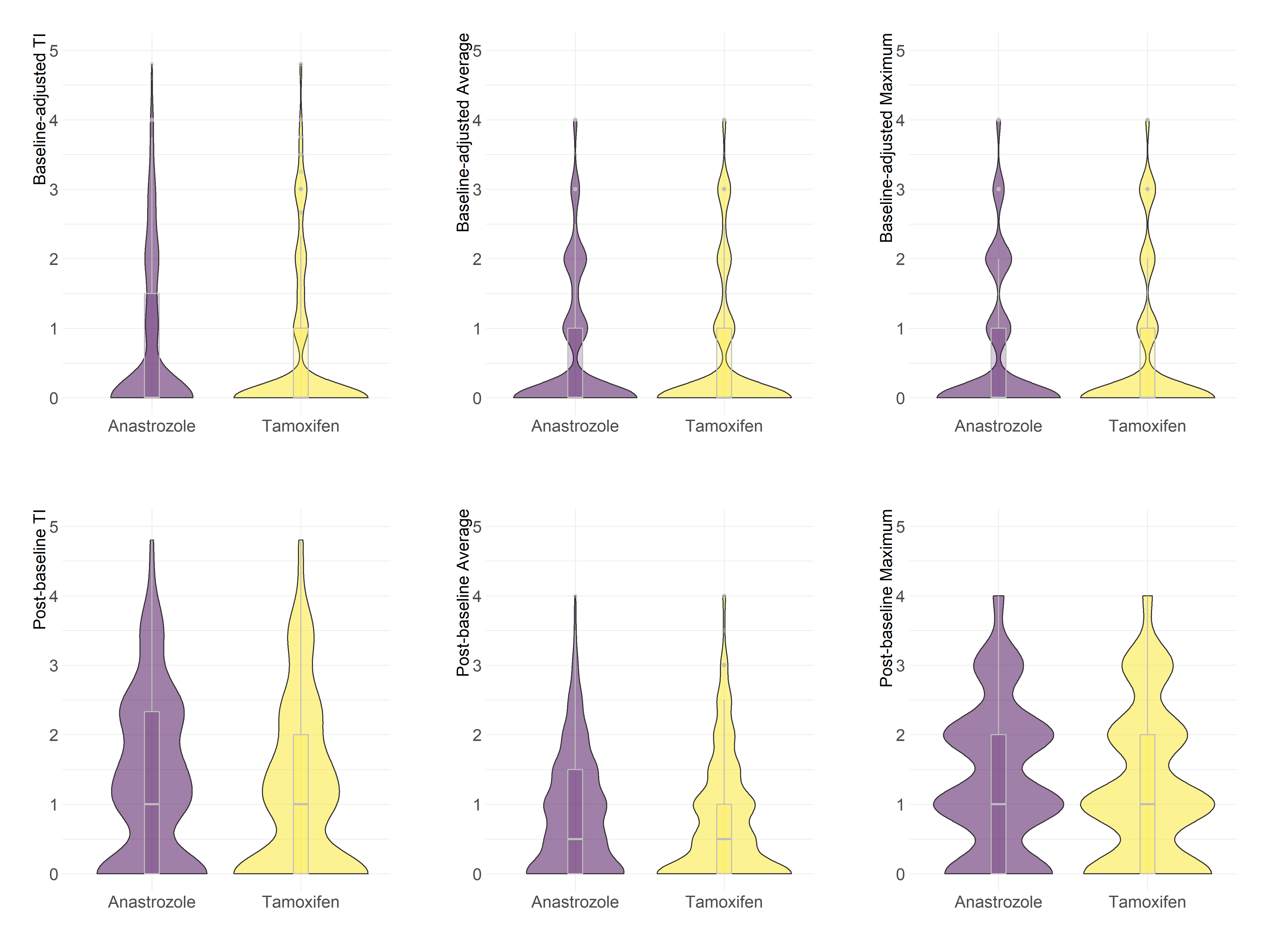


**Fig. 7** Violin plot of headache score from the B-35 trial summarized in a single measure by each method.

A violin plot [2] is a method of plotting numeric data which depicts distributions of numeric data for one or more groups using the boxplot and probability density function (PDF), which is a rotated, centered, and smoothed histogram. It has the same summary statistics as boxplot like the median, interquartile range (IQR), outliers using a method that is function of the IQR. In addition, the PDF estimation shows the distribution shape of the data. A narrower PDF represents that the value occurs less frequently, while wider indicates that the value occurs more frequently.

In the B-35 trial [3,4], higher headache score was observed for anastrozole arm when the post-baseline method is used to summarize the scores into a single value, while for the baseline-adjusted method, the summary measure distribution shape was close in both treatment.

**5 Simulation Study**

**Algorithm to generate the dataset and performing the simulation study**

**Step 1:** Define the sample size in each group and the number of measures over time.

Comments: 100 subjects per group and three time points (baseline, time 2, and time 3) were considered in our simulation study.

**Step 2:** For each group and time point, set the expected proportion in each category of the response variable (with five categories), such their sum is one.

An example is illustrated as follows:

**
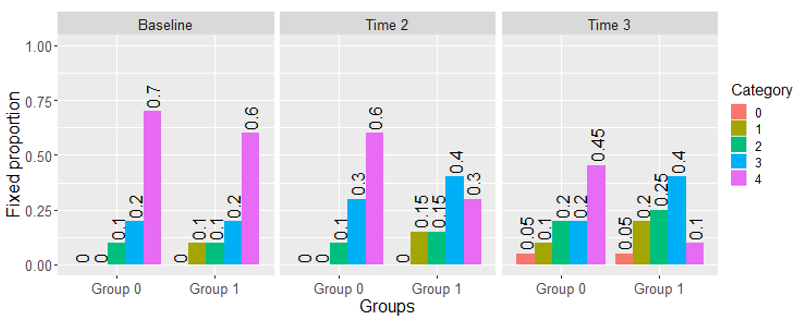
**

**Fig. 8** Fixed proportions in each category for a single item longitudinal.

Comments: At baseline, the probability of occurrence of grades 0, 1, 2, 3, 4 (category 0 to 4) were set to 0, 0, 0.1, 0.2, and 0.7 in Group 0, and 0, 0.1, 0.1, 0.2, and 0.6 in Group 1, respectively. Different values were fixed for the marginal probabilities in time 2 and time 3 in each group. In this example, the assumption is that the groups are different in all time points. Figures 1-6 show all the fixed probabilities that were used to generate the datasets in our simulation study.

**Step 3:** The desired correlation value among the repeated measures.

Comments: We fixed three different correlation values among time points (ρ = 0.2, 0.5, and 0.9) in our simulation study.

**Step 4:** With the fixed parameter values (Steps 1-3) generate the random sample of the multinomial copula model [5].

An example of generated dataset is shown below:

| 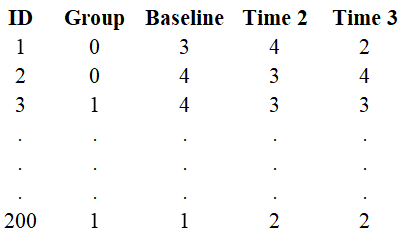 | 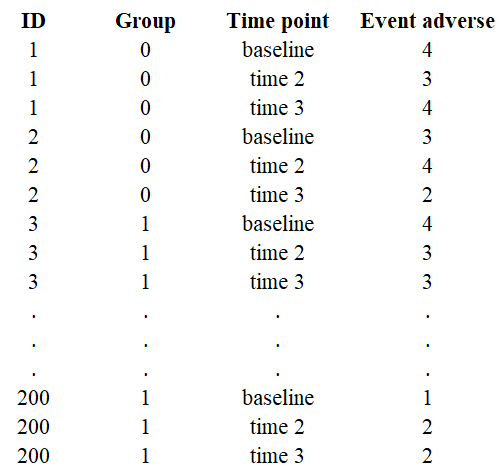 |
| --- | --- |

**Fig. 9** Example of a dataset generated according to the multinomial copula model using the fixed parameter values in Steps 1-3 (left panel: wider format; right panel: long format) assuming 100 subjects in each group and ρ = 0.5 among the repeated measures.

Comments: For example, the subject 1 is in the group 0 and reported Grade 3 at baseline, Grade 4 in time 2, and Grade 2 in time 3. In the generated dataset, it is expected to observe 10%, 20%, and 70% of Grades 2, 3 and 4, respectively, at baseline for the Group 0, as defined in Step 2 (Fig 8).

**Step 5:** To apply the methods for summarizing toxicity over time in a single measure using the baseline-adjusted and post-baseline methods with the summary measures TI, Avg, and Max. The calculation of these summary measures were examplified in Section 2 - Tables S2 and S3.

An example of generated dataset considering the single measures using the baseline-adjusted and post-baseline scores is shown below:

**
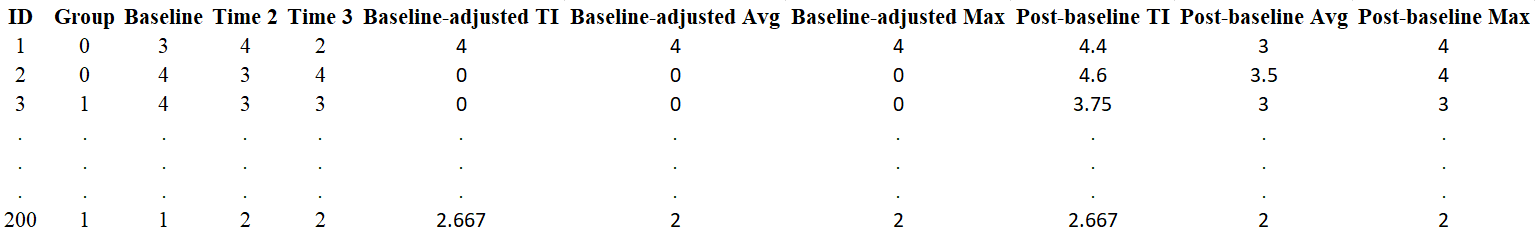
**

**Fig. 10** Example of dataset generated according to the multinomial copula model using the fixed parameter values in Steps 1-3 and the toxicity over time summarized in a single measure using the baseline-adjusted and post-baseline TI, Avg, and Max scores.

Comments: Patient 1 reported Grade 3 at baseline, Grade 4 in time 2, and Grade 2 in time 3. The toxicity summarized in a single measure using the baseline-adjusted TI, Avg, Max was 4, 4, and 4, respectively, and for the post-baseline TI, Avg and Max was 4.4, 3, and 4, respectively.

**Step 6:** Fit the PIM considering each summary measure computed in Step 5 as response variable and the group variable as a covariate. In addition, for the post-baseline score consider the baseline measurement also as a covariate in the modeling (this analysis method was denoted throughout in article as PIM baseline as covariate TI, Avg, and Max depending on summary measure used, respectively).

An R user can run the PIMs using the dataset in the wider format as below:

library(pim)

fit1 <- pim(Baseline-adjusted TI~ Group, data = dataset)

fit2 <- pim(Baseline-adjusted Avg~ Group, data = dataset)

fit3 <- pim(Baseline-adjusted Max~ Group, data = dataset)

fit4 <- pim(Baseline-adjusted TI~ Baseline+Group, data = dataset)

fit5 <- pim(Baseline-adjusted Avg~ Baseline+Group, data = dataset)

fit6 <- pim(Baseline-adjusted Max~ Baseline+Group, data = dataset)

**Step 7:** Fit the cumulative logit link model (CLMM) considering the measures in time points 2 and 3 as response variable and the baseline measurement and group as covariates (fixed effects).

An R user can run the CLMM using the dataset in the long format as below:

library(ordinal)

fit7<-clmm(Event adverse~Group+Time point+Baseline+(1|ID),data=dataset)

The CLMM (Bootstrap) statistical analysis method is performed fitting the CLMM as shown in above. However, the p-value associated with the group effect is obtained by using the likelihood ratio test (LRT) by parametric bootstrap with B=500 bootstrap resamples as previsouly detailed in Section 3.

**Step 8:** For each generated dataset, the comparison between groups was performed using the following eight statistical analysis methods: PIM Baseline-adjusted TI (fit1), Avg (fit2), and Max (fit3); PIM Baseline as covariate TI (fit4), Avg (fit5), and Max (fit6); CLMM (fit7) and CLMM (Bootstrap) (fit7: using the LRT by parametric bootstrap approach).

For each scenario, generate $n_{sim}$ independent replicates (independent datasets) to estimate the type I and II error rates. The type I error rate is estimated as the proportion of rejection of the null hypothesis ($H_{0}$) when it is true, i.e., when there is not difference between arms, and the power is computed as the proportion of rejection of the $H_{0}$ when it is false, i.e., when the two groups are different over time.

Comments: We considered $n_{sim}$=1000 for each scenario and the type I and II error rates were obtained based on 1000 p-values derived of the Wald statistic, LRT or LRT by parametric bootstrap. An appropriate test would reject a proportion of α=0.05 (significance level) of the 1000 repetitions when the null hypothesis is true (i.e., when there is not difference between groups). If the test does not control type I error at level 𝛼, power should be interpreted with caution. In our simulation study, the type I error rates were obtained for each statistical analysis method based on 45 scenarios (15 scenarios with low (ρ=0.2), moderate (ρ=0.5), and high (ρ=0.9) correlation among the repeated measures). Fixed proportions of the categories in each time point by group to generate the datasets under the assumption the groups are equal over time are reported in Figure 6. Under the assumption the groups are different, 225 scenarios were considered, and the power of each statistical analysis method was summarized using the average of the 225 power estimates. In addition, for each statistical analysis method, the empirical powers were plotted as one minus the empirical cumulative distribution function (1-ECDF). The fixed proportions of the categories in each time point by group to generate the datasets under the assumption the groups are different are reported in Figures 1-5.

**Results**

Figure 11 illustrates the power estimates comparisons among the statistical analysis methods according to the simulated data type as described in Table S1. The y-axis can be interpreted as the probability of a given statistical analysis method to have power greater than a given threshold and the x-axis represents possible thresholds based on the power estimates. Curves at the top indicate that a larger number of scenarios showed power greater than a given threshold than curves at the bottom.


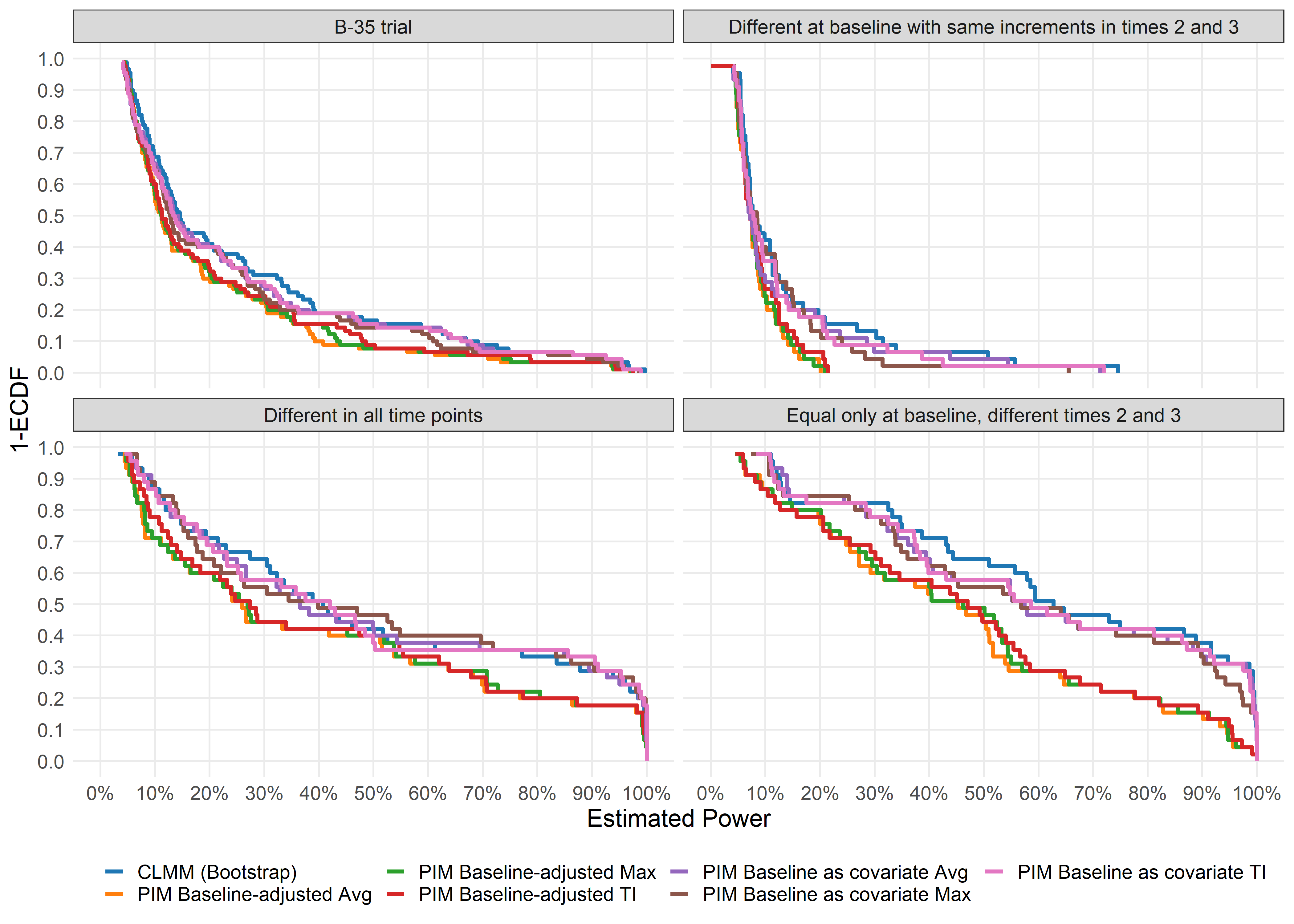
 **Fig. 11** One minus the cumulative distribution function of the power estimates by method in each simulated data type under the assumption that the groups are different.

**Table S4** Power estimates according to the simulated data type by analysis method

| **Statistical analysis method** |  | **B-35 trial*** |  |  | |  |
| --- | --- | --- | --- | --- | --- | --- |
|  |  | **Power#** | **95%CI of power** | **Mean difference (MD)** | | **95%CI of MD** |
| PIM Baseline-adjusted Max | | 20.52% | 16.13% to 24.91% | 6.11% | | 4.59% to 7.64% |
| PIM Baseline-adjusted Avg | | 19.91% | 15.62% to 24.20% | 6.72% | | 5.00% to 8.44% |
| PIM Baseline-adjusted TI | | 21.22% | 16.67% to 25.77% | 5.41% | | 4.11% to 6.72% |
| PIM Baseline as covariate Max | | 24.03% | 18.92% to 29.14% | 2.60% | | 1.88% to 3.33% |
| PIM Baseline as covariate Avg | | 25.27% | 19.96% to 30.58% | 1.36% | | 0.68% to 2.05% |
| PIM Baseline as covariate TI | | 25.11% | 19.80% to 30.42% | 1.52% | | 0.84% to 2.22% |
| CLMM (Bootstrap) | | 26.63% | 21.30% to 31.96% | Ref | | Ref |
|  |  | **Different in all time points**** | |  |  |  |
|  |  | **Power#** | **95%CI of power** | **Mean difference (MD)** | | **95%CI of MD** |
| PIM Baseline-adjusted Max | | 41.50% | 30.78% to 52.22% | 9.22% | | -3.18% to 21.63% |
| PIM Baseline-adjusted Avg | | 40.92% | 30.24% to 51.60% | 9.80% | | -2.54% to 22.15% |
| PIM Baseline-adjusted TI | | 42.20% | 31.66% to 52.74% | 8.52% | | -3.69% to 20.73% |
| PIM Baseline as covariate Max | | 50.93% | 39.54% to 62.32% | -0.21% | | -5.58% to 5.15% |
| PIM Baseline as covariate Avg | | 50.42% | 39.23% to 61.61% | 0.30% | | -0.99% to 1.60% |
| PIM Baseline as covariate TI | | 50.31% | 39.06% to 61.56% | 0.41% | | -1.76% to 2.58% |
| CLMM (Bootstrap) | | 50.72% | 39.76% to 61.68% | Ref | | Ref |
|  |  | **Equal only at baseline, different times 2 and 3**** | | |  |  |
|  |  | **Power#** | **95%CI of power** | **Mean difference (MD)** | | **95%CI of MD** |
| PIM Baseline-adjusted Max | | 46.72% | 37.65% to 55.79% | 16.99% | | 10.12% to 23.86% |
| PIM Baseline-adjusted Avg | | 45.78% | 36.77% to 54.79% | 17.93% | | 10.75% to 24.90% |
| PIM Baseline-adjusted TI | | 47.20% | 38.03% to 56.37% | 16.51% | | 9.82% to 23.19% |
| PIM Baseline as covariate Max | | 59.73% | 49.69% to 69.77% | 3.98% | | 0.86% to 7.10% |
| PIM Baseline as covariate Avg | | 60.84% | 50.78% to 70.90% | 2.87% | | 0.77% to 4.98% |
| PIM Baseline as covariate TI | | 60.90% | 50.70% to 71.10% | 2.81% | | 0.65% to 4.97% |
| CLMM (Bootstrap) | | 63.71% | 53.69% to 73.73% | Ref | | Ref |
|  |  | **Different at baseline with same increments in times 2 and 3**** | | |  |  |
|  |  | **Power#** | **95%CI of power** | **Mean difference (MD)** | | **95%CI of MD** |
| PIM Baseline-adjusted Max | | 8.44% | 7.17% to 9.71% | 5.78% | | 1.49% to 10.07% |
| PIM Baseline-adjusted Avg | | 8.23% | 7.00% to 9.46% | 5.99% | | 1.66% to 10.32% |
| PIM Baseline-adjusted TI | | 8.85% | 7.42% to 10.28% | 5.37% | | 1.07% to 9.67% |
| PIM Baseline as covariate Max | | 11.89% | 8.69% to 15.09% | 2.33% | | -0.52% to 5.18% |
| PIM Baseline as covariate Avg | | 12.69% | 8.56% to 16.82% | 1.53% | | 0.56% to 2.50% |
| PIM Baseline as covariate TI | | 12.47% | 8.70% to 16.24% | 1.75% | | 0.20% to 3.30% |
| CLMM (Bootstrap) | | 14.22% | 9.81% to 18.63% | Ref | | Ref |

^#^Mean power estimates obtained under area 1-ECFD curve. Mean difference (MD) represents the mean difference of power estimates by CLMM compared to other statistical analysis methods. 95%CI was computed using the paired t-test considering the sample of power estimates. 95%CIs that exclude zero indicate statistically significant differences at the 5% significance level (two-sided hypothesis test). *Based on 90 simulated scenarios. **Based on 45 simulated scenarios.

**Table S5** Mean difference (MD) of power estimates by PIM Baseline as covariate compared to PIM Baseline-adjusted statistical analysis method by simulated scenarios.

| **Statistical analysis method** |  | **Overall*** |  |  | **B-35 trial***** |  |
| --- | --- | --- | --- | --- | --- | --- |
|  |  | **Mean difference (MD)** | **95%CI of MD** |  | **Mean difference (MD)** | **95%CI of MD** |
| PIM Baseline-adjusted Max |  | Ref |  |  | Ref |  |
| PIM Baseline as covariate Max |  | 6.58% | 3.97% to 9.20% |  | 3.51% | 2.21% to 4.82% |
|  |  |  |  |  |  |  |
| PIM Baseline-adjusted Avg |  | Ref |  |  | Ref |  |
| PIM Baseline as covariate Avg |  | 7.95% | 4.95% to 10.94% |  | 5.36% | 3.53% to 7.18% |
|  |  |  |  |  |  |  |
| PIM Baseline-adjusted TI |  | Ref |  |  | Ref |  |
| PIM Baseline as covariate TI |  | 6.64% | 3.81% to 9.47% |  | 3.89% | 2.49% to 5.29% |
|  |  | **ρ=0.2**** |  |  | **Different in all time points^#^** |  |
|  |  | **Mean difference (MD)** | **95%CI of MD** |  | **Mean difference (MD)** | **95%CI of MD** |
| PIM Baseline-adjusted Max | | Ref |  |  | Ref |  |
| PIM Baseline as covariate Max | | 8.86% | 3.69% to 14.02% |  | 9.43% | -1.24% to 20.11% |
|  |  |  |  |  |  |  |
| PIM Baseline-adjusted Avg | | Ref |  |  | Ref |  |
| PIM Baseline as covariate Avg | | 11.12% | 5.34% to 16.90% |  | 9.50% | -2.64% to 21.65% |
|  |  |  |  |  |  |  |
| PIM Baseline-adjusted TI | | Ref |  |  | Ref |  |
| PIM Baseline as covariate TI | | 9.43% | 4.03% to 14.84% |  | 8.11% | -3.51% to 19.72% |
|  |  | **ρ=0.5**** |  |  | **Equal only at baseline, different times 2 and 3^#^** |  |
|  |  | **Mean difference (MD)** | **95%CI of MD** |  | **Mean difference (MD)** | **95%CI of MD** |
| PIM Baseline-adjusted Max | | Ref |  |  | Ref |  |
| PIM Baseline as covariate Max | | 6.69% | 2.51% to 10.88% |  | 13.01% | 6.44% to 19.59% |
|  |  |  |  |  |  |  |
| PIM Baseline-adjusted Avg | | Ref |  |  | Ref |  |
| PIM Baseline as covariate Avg | | 8.14% | 3.58% to 12.68% |  | 15.06% | 7.80% to 22.32% |
|  |  |  |  |  |  |  |
| PIM Baseline-adjusted TI | | Ref |  |  | Ref |  |
| PIM Baseline as covariate TI | | 6.96% | 2.66% to 11.26% |  | 13.70% | 6.70% to 20.69% |
|  |  | **ρ=0.9**** |  |  | **Different at baseline with same increments in times 2 and 3^#^** |  |
|  |  | **Mean difference (MD)** | **95%CI of MD** |  | **Mean difference (MD)** | **95%CI of MD** |
| PIM Baseline-adjusted Max | | Ref |  |  | Ref |  |
| PIM Baseline as covariate Max | | 4.20% | -0.12% to 8.60% |  | 3.45% | 0.20% to 6.70% |
|  |  |  |  |  |  |  |
| PIM Baseline-adjusted Avg | | Ref |  |  | Ref |  |
| PIM Baseline as covariate Avg | | 4.59% | -0.70% to 9.86% |  | 4.46% | 0.33% to 8.59% |
|  |  |  |  |  |  |  |
| PIM Baseline-adjusted TI | | Ref |  |  | Ref |  |
| PIM Baseline as covariate TI | | 3.53% | -1.55% to 8.60% |  | 3.69% | -0.17% to 7.41% |

Mean difference (MD) represents the mean difference of power estimates by PIM Baseline as covariate compared to the PIM Baseline-adjusted statistical analysis method. 95%CI was computed using the paired t-test considering the sample of power estimates. 95%CIs that exclude zero indicate statistically significant differences at the 5% significance level (two-sided hypothesis test). *Based on 225 simulated scenarios. **Based on 75 simulated scenarios. ***Based on 90 simulated scenarios. ^#^Based on 45 simulated scenarios.

**References**

1. Luke, S. G. (2017). Evaluating significance in linear mixed-effects models in R. Behavior Research Methods, 49(4), 1494-1502. doi: 10.3758/s13428-016-0809-y
2. Hintze, J. L., & Nelson, R. D. (1998). Violin plots: a box plot-density trace synergism. The American Statistician, 52(2), 181-184.
3. Margolese, R.G., R.S. Cecchini, T.B. Julian, P.A. Ganz, J.P. Costantino, L.A. Vallow, K.S. Albain, P.W. Whitworth, M.E. Cianfrocca, A.M. Brufsky, H.M. Gross, G.S. Soori, J.O. Hopkins, L. Fehrenbacher, K. Sturtz, T.F. Wozniak, T.E. Seay, E.P. Mamounas and N. Wolmark. (2016). Anastrozole versus tamoxifen in postmenopausal women with ductal carcinoma in situ undergoing lumpectomy plus radiotherapy (NSABP B-35): a randomised, double-blind, phase 3 clinical trial. Lancet. 387(10021): 849-56.<https://doi.org/10.1016/S0140-6736(15)01168-X>
4. Forbes, J.F., I. Sestak, A. Howell, B. Bonanni, N. Bundred, C. Levy, G. von Minckwitz, W. Eiermann, P. Neven, M. Stierer, C. Holcombe, R.E. Coleman, L. Jones, I. Ellis, J. Cuzick and I.-I. investigators. (2016). Anastrozole versus tamoxifen for the prevention of locoregional and contralateral breast cancer in postmenopausal women with locally excised ductal carcinoma in situ (IBIS-II DCIS): a double-blind, randomised controlled trial. Lancet. 387(10021): 866-73.<https://doi.org/10.1016/S0140-6736(15)01129-0>
5. Razaee, Z., & Amini, A. (2020). The Potts-Ising model for discrete multivariate data. Advances in Neural Information Processing Systems, 33.
